# Strain-Specific Variation of the Decorin-Binding Adhesin DbpA Influences the Tissue Tropism of the Lyme Disease Spirochete

**DOI:** 10.1371/journal.ppat.1004238

**Published:** 2014-07-31

**Authors:** Yi-Pin Lin, Vivian Benoit, Xiuli Yang, Raúl Martínez-Herranz, Utpal Pal, John M. Leong

**Affiliations:** 1 Department of Molecular Biology and Microbiology, Tufts University School of Medicine, Boston, Massachusetts, United States of America; 2 Department of Veterinary Medicine, University of Maryland, College Park, Maryland, United States of America; 3 Virginia–Maryland Regional College of Veterinary Medicine, College Park, Maryland, United States of America; University of Montana, United States of America

## Abstract

Lyme disease spirochetes demonstrate strain- and species-specific differences in tissue tropism. For example, the three major Lyme disease spirochete species, *Borrelia burgdorferi* sensu stricto, *B. garinii*, and *B. afzelii*, are each most commonly associated with overlapping but distinct spectra of clinical manifestations. *Borrelia burgdorferi* sensu stricto, the most common Lyme spirochete in the U.S., is closely associated with arthritis. The attachment of microbial pathogens to cells or to the extracellular matrix of target tissues may promote colonization and disease, and the Lyme disease spirochete encodes several surface proteins, including the decorin- and dermatan sulfate-binding adhesin DbpA, which vary among strains and have been postulated to contribute to strain-specific differences in tissue tropism. DbpA variants differ in their ability to bind to its host ligands and to cultured mammalian cells. To directly test whether variation in *dbpA* influences tissue tropism, we analyzed murine infection by isogenic *B. burgdorferi* strains that encode different *dbpA* alleles. Compared to *dbpA* alleles of *B. afzelii* strain VS461 or *B. burgdorferi* strain N40-D10/E9, *dbpA* of *B. garinii* strain PBr conferred the greatest decorin- and dermatan sulfate-binding activity, promoted the greatest colonization at the inoculation site and heart, and caused the most severe carditis. The *dbpA* of strain N40-D10/E9 conferred the weakest decorin- and GAG-binding activity, but the most robust joint colonization and was the only *dbpA* allele capable of conferring significant joint disease. Thus, *dbpA* mediates colonization and disease by the Lyme disease spirochete in an allele-dependent manner and may contribute to the etiology of distinct clinical manifestations associated with different Lyme disease strains. This study provides important support for the long-postulated model that strain-specific variations of *Borrelia* surface proteins influence tissue tropism.

## Author Summary

Lyme disease, the most common vector-borne disease in the United States, is caused by a bacterium, *Borrelia burgdorferi*. This bacterium infects the skin at the site of the tick bite and then can spread to other tissues, such as the heart, joints or nervous system, causing carditis, arthritis or neurologic disease. To colonize human tissues, the pathogen produces surface proteins that promote bacterial attachment to these sites. For example, DbpA binds to decorin, a component of human tissue. Different Lyme disease strains differ in the particular tissues they colonize and the disease they cause, but we do not understand why. Different strains also make distinct versions of DbpA that bind decorin differently, so variation of DbpA might contribute to strain-to-strain variation in clinical manifestations. To test this, we infected mice with Lyme disease strains that were identical except for the particular DbpA variant they produced. We found that the strains colonized different tissues and caused different diseases, such as arthritis or carditis. These results provide the first solid evidence that variation of an outer surface protein, in this case DbpA, influences what tissues are most affected during Lyme disease.

## Introduction

Lyme disease is distributed worldwide and is the most common arthropod-borne infectious disease in the United States [1]–[3]. The causative agent is the spirochete *Borrelia burgdorferi* sensu lato, which includes *B. burgdorferi* sensu stricto, *B. garinii*, and *B. afzelii* [4] [5]. Following the bite of an infected *Ixodes* tick, the Lyme disease spirochete produces a local infection, resulting in the characteristic skin lesion erythema migrans. In the absence of antibiotic treatment, spirochetes may disseminate to multiple organs, including joints, the central nervous system, and the heart, resulting in diverse manifestations such as arthritis, neurological abnormalities, and carditis [2], [6]–[9].

Lyme disease spirochetes demonstrate strain- and species-specific differences in tissue tropism. For example, *B. burgdorferi* sensu stricto, most prevalent in the United States, *B. garinii* and *B. afzelii*, each more common in Europe [1], [5], are genetically distinct and are associated with different typical chronic manifestations: *B. burgdorferi* with arthritis, *B. garinii* with neuroborreliosis, and *B. afzelii* with the chronic skin lesion acrodermatitis [10]. In addition, the severity of human symptoms and the dissemination activities of different strains within a single Lyme disease species may also differ significantly [9], [11], [12]. Strain-to-strain variation in dissemination and disease manifestation has also been observed in animal studies [13], [14].

The basis for differences in tissue tropism and/or disease severity is not well understood. Several documented or putative virulence factors encoded by Lyme disease spirochete vary in a strain-specific manner [3], [15]–[17]. In some instances this variation is associated with differences in the postulated biological activity of the factor, e.g. binding of complement regulators by CspZ and other CRASPs (complement regulator-acquiring surface proteins) or binding of plasminogen by the outer surface protein OspC [16]–[19]. Moreover, in a set of three Lyme disease strains, invasiveness correlated with the ability of OspC to bind plasminogen [18], [20], giving rise to the hypothesis that allelic variation of *B. burgdorferi* surface proteins have the capacity to contribute to tissue tropism of different Lyme disease spirochete strains [9], [12], [18], [21]. However, to date rigorous demonstration that isogenic strains harboring allelic variants of virulence genes indeed behave differently during animal infection has been lacking.

Adhesion of bacterial pathogens to host cells or extracellular matrix (ECM) of target tissues, often mediated by outer surface protein adhesins, is thought to be an important early step in tissue colonization [22]. In fact, *Borrelia* sp. encode a plethora of adhesins that have been found to recognize different ECM components and/or to promote binding to diverse mammalian cell types [23]–[25]. Two related *Borrelia* adhesins, decorin binding proteins A and B (DbpA and DbpB, respectively), encoded by a bicistronic operon [26], bind to both decorin and to the glycosaminoglycan (GAG) dermatan sulfate [27], [28]. Whereas the DbpB sequence is highly conserved in different strains of *B. burgdorferi* sensu lato, the DbpA sequence is highly polymorphic, with sequence similarities as low as 58% between variants [29].

Spirochetes disseminate less efficiently in decorin-deficient compared to wild type mice, suggesting an important function for decorin binding in spirochete tissue spread. [30]. *B. burgdorferi* lacking DbpA and DbpB in fact exhibited both reduced colonization and dissemination activity and a three- to four-log increase in ID_50_, indicating that these adhesins play a significant role in infection [31]–[35]. Consistent with this role, *dbpA* and *dbpB* are expressed efficiently in culture conditions that may reflect the host environment, such as at mammalian body temperature or in the presence of atmospheric CO_2_ [36]–[38].

The ability of DbpA to bind to decorin and/or dermatan sulfate requires an intact C-terminus, and DbpA variants demonstrate differences in decorin- and/or dermatan sulfate-binding activities [21], [39]. Given the abovementioned strain- and species-specific differences in tissue tropism among Lyme disease spirochetes, an attractive hypothesis is that the decorin and/or GAG-binding activities of DbpA (and DbpB) are critical for promoting colonization, and that allelic variation of *dbpA* might influence the tissue tropism of Lyme disease spirochetes. In the current study, we infected mice with various isogenic *B. burgdorferi* strains encoding DbpA variants, or a non-binding mutant. These studies indicate that decorin- and/or GAG-binding activity of DbpA is required for colonization functions. Importantly we also found that allelic variation of *dbpA* contributes to differences in tissue tropism.

## Results

### Recombinant DbpA protein variants show differences in binding to decorin and dermatan sulfate

We previously tested the ability of DbpA mutants or variants to mediate binding of a non-adhesive and non- infectious *B. burgdorferi* strain to decorin, dermatan sulfate or mammalian cells [39]. DbpA_VS461_ΔC11, which lacks the 11 C-terminal residues of DbpA_VS461_, was shown to be unable to promote spirochetal binding to decorin or dermatan sulfate. In addition, a set of variants that included DbpA from *B. burgdorferi* strains B31 (DbpA_B31_), 297 (DbpA_297_), N40-D10/E9 (DbpA_N40-D10/E9_), B356 (DbpA_B356_), *B. afzelii* VS461 (DbpA_VS461_), and *B. garinii* PBr (DbpA_PBr_), showed variant-specific differences in the ability to promote bacterial adhesion to the two substrates. By using semi-quantitative ELISA, this study also analyzed the binding of recombinant versions of DbpA variants except DbpA_297_ and DbpA_B356_, which display 90% and 99% similarities to DbpA_B31_ and DbpA_N40-D10/E9_, respectively. To measure the decorin- and dermatan sulfate-binding affinities of DbpA variants more precisely, here we utilized quantitative ELISA and surface plasmon resonance (SPR; Fig. S2A and Table 1). The two independent methods for assessing binding gave results entirely consistent with each other and revealed dissociation constants indicating (1) robust decorin-binding by DbpA_PBr_ (K_D_ = 0.06–0.09 µM); (2) moderate decorin-binding by DbpA_B31_, DbpA_297_, and DbpA_VS461_ (K_D_ = 0.14–0.30 µM); (3) less efficient decorin-binding by DbpA_N40-D10/E9_ and DbpA_B356_ (K_D_ = 0.71-0.95 µM). Interestingly, a BXBB motif (residues 64 to 67) that has been proposed to form a positively charged pocket that binds to decorin and/or dermatan sulfate [40], is not found in DbpA_PBr_ (Fig. S6), suggesting that BXBB is not essential for decorin- or dermatan sulfate-binding.

**Table 1:** DbpA variants differ in binding to decorin and dermatan sulfate.

		ELISA	----- Surface Plasmon Resonance -----		
DbpA variant	Ligand	K_D_ (µM)	K_D_ (µM)	k_on_ (10^5^ s^−1^M^−1^)	k_off_ (s^−1^)
***B. burgdorferi***					
**DbpA_B31_**	**Decorin**	**0.21±0.03**	**0.28±0.03**	4.17±0.70	0.11±0.01
	**Derm SO_4_**	**0.91±0.02**	**0.63±0.04**	0.36±0.01	0.02±0.01
**DbpA_297_**	**Decorin**	**0.14±0.06**	**0.30±0.08**	3.51±0.86	0.10±0.01
	**Derm SO_4_**	**0.78±0.18**	**0.50±0.01**	0.52±0.02	0.02±0.01
**DbpA_B356_**	**Decorin**	**0.95±0.12**	**0.84±0.04**	0.66±0.33	0.06±0.01
	**Derm SO_4_**	**3.68±0.21**	**1.91±0.87**	0.44±0.04	0.07±0.03
**DbpA_N40-D10/E9_**	**Decorin**	**0.85±0.12**	**0.71±0.01**	0.97±0.42	0.07±0.01
	**Derm SO_4_**	**3.10±0.26**	**2.58±0.17**	0.13±0.06	0.03±0.01
***B. garinii***					
**DbpA_PBr_**	**Decorin**	**0.06±0.01**	**0.09±0.06**	18.3±1.10	0.13±0.02
	**Derm SO_4_**	**0.21±0.03**	**0.16±0.02**	0.56±0.02	0.009±0.001
***B. afzelii***					
**DbpA_VS461_**	**Decorin**	**0.29±0.07**	**0.26±0.02**	1.52±0.23	0.04±0.01
	**Derm SO_4_**	**1.55±0.63**	**1.61±0.48**	0.43±0.06	0.07±0.03
**DbpA_VS461_ΔC11**	**Decorin**	**n.b** a	**n.b.**	n.b.	n.b.
	**Derm SO_4_**	**n.b.**	**n.b.**	n.b.	n.b.

All values represent the mean ± SEM of three experiments.

a No binding activity was detected.

With the exception of DbpA_VS461_, the calculated K_D_ for dermatan sulfate binding of each DbpA variant was approximately two- to four-fold higher than its K_D_ for decorin binding; DbpA_VS461_ bound to dermatan sulfate approximately six-fold less efficiently than to decorin (Fig. S2 and Table 1). Finally, recombinant protein DbpA_VS461_ΔC11, which was found by far-UV CD analysis (Fig. S1) to retain the secondary structure of wild-type DbpA_VS461_, was unable to bind to decorin or dermatan sulfate. These findings were entirely consistent with previous results determined with less quantitative methods [39], and likely reflect the fact that sequence lacking in DbpA_VS461_ΔC11 includes conserved K170, a lysine residue previously shown to be critical for decorin-binding activity [41] (Fig. S6).

### DbpA variants alter the ability of an infectious strain of *B. burgdorferi* to bind decorin and dermatan sulfate

DbpA_PBr_, DbpA_VS461_, and DbpA_N40-D10/E9_ each represent one of the three binding profiles described above, as well as collectively encompass the three major genospecies of Lyme disease spirochetes, each of which has been associated with different human clinical manifestations. To focus on how variations in DbpA binding to decorin and dermatan sulfate may influence the infectious process and avoid potential functional redundancy associated with the production of another decorin- and dermatan sulfate-binding adhesin, we generated DbpA-producing strains that did not produce DbpB. We generated a set of plasmids that encode the *bbe22* gene, which is required for spirochete survival in a mammalian host [42], and the coding region of *dbpA_PBr_*, *dbpA_VS461_*, or *dbpA_N40-D10/E9_*, or *dbpA_VS461_*Δ*C11* (as a non-binding control) under the control of the *dbpBA* promoter of *B. burgdorferi* strain B31. The plasmids encoding different *dbpA* alleles were then individually introduced into a *dbpBA* deletion mutant of the highly transformable infectious strain *B. burgdorferi* ML23, a derivative of *B. burgdorferi* B31 that lacks *bbe22* and therefore cannot survive in the mouse in the absence of a complementing *bbe22*-encoding plasmid [43]. We verified by flow cytometry analysis that the DbpA variants produced in *B. burgdorferi* ML23Δ*dbpBA* were located on the surface of the recombinant spirochetes, and at levels indistinguishable from that of their DbpA-proficient parental strain ML23 (Fig. S3).

**Figure 1:**
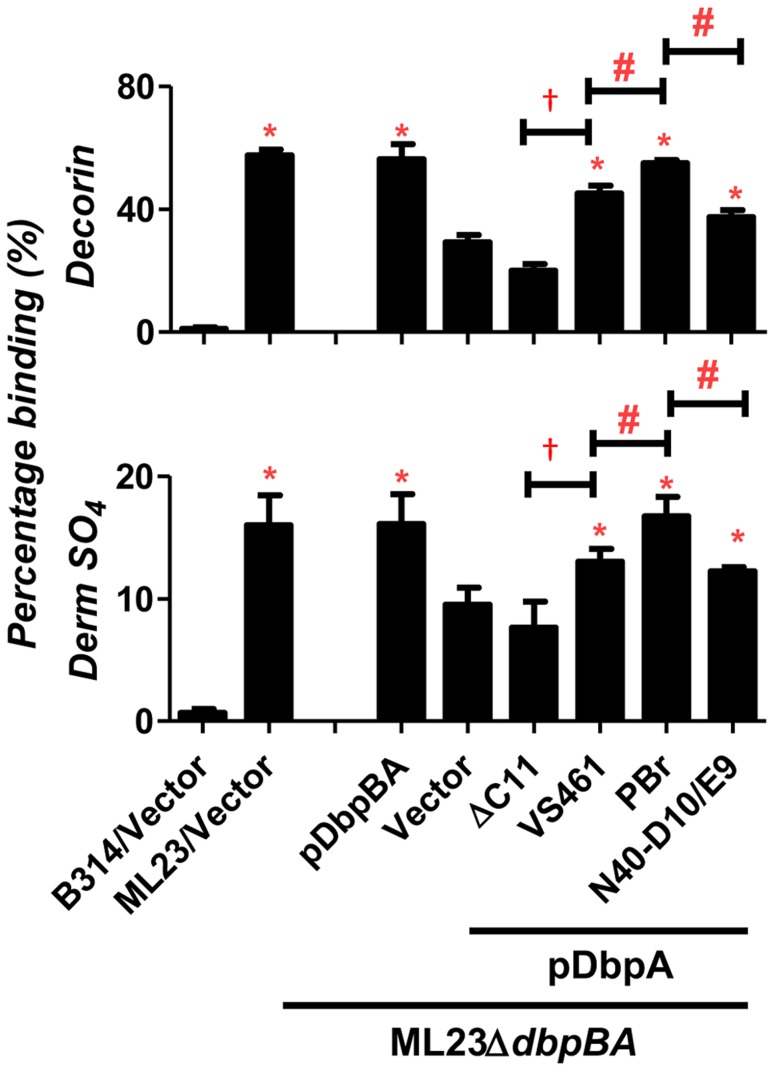
DbpA variants produced in *B. burgdorferi* promote distinct binding activities to decorin and dermatan sulfate. Binding of radiolabeled *B. burgdorferi* ML23/pBBE22, ML23Δ*dbpBA*/pBBE22 (“Vector”), and the deletion strain bearing a plasmid encoding DbpA and DbpB (“pDbpBA”), DbpA_VS461_ΔC11 (“ΔC11”), DbpA_VS461_ (“VS461”), DbpA_PBr_ (“PBr”) or DbpA_N40-D10/E9_ (“N40-D10/E9”), to decorin, dermatan sulfate (Derm SO_4_), or the negative control chondroitin-6-sulfate (see Materials and Methods). The non-adherent *B. burgdorferi* strain B314 harboring the empty vector pJF21 (“B314/Vector”) was also included as negative control. The percentage of bound bacteria was determined by radioactive counts in bound bacteria normalized to the counts in the inoculum.Each bar represents the mean of four independent determinations ± SEM. Statistical significance was determined using the one-way ANOVA test. Significant (P<0.05) differences in binding relative to the *dbpBA* deletion strain (“*”), between two strains relative to each other (“#”), or relative to the *dbpA_VS461_ΔC11*-complemented strain (“†”) are indicated.

We next investigated the distinct decorin- and dermatan sulfate-binding activities specifically conferred to infectious strain ML23 by the various *dbpA* alleles. We measured binding of radiolabeled ML23Δ*dbpBA* strains producing DbpA variants to microtiter wells coated with decorin or dermatan sulfate. Chondroitin-6-sulfate, included as a negative control, mediated binding of less than 5% of inoculum (data not shown). Strain ML23 harboring the vector alone, a positive control that expresses both DbpA and DbpB, bound to decorin or dermatan sulfate with an efficiency of approximately 55% or 15%, respectively (Fig. 1). This level of binding was significantly greater than binding by strain ML23Δ*dbpBA* harboring vector alone, i.e. approximately 30% or 10% for binding to decorin or dermatan sulfate, respectively (Fig. 1). This “background” (i.e., DbpB- and DbpA-independent) decorin- and dermatan sulfate-binding activity of strain ML23Δ*dbpBA* is considerably greater than that of the high-passage strain *B. burgdorferi* B314 (i.e., less than 2% for either substrate), suggesting that decorin- and dermatan sulfate-binding adhesins other than DbpA and DbpB are expressed by strain ML23Δ*dbpBA*. As expected, the production of both DbpA and DbpB in strain ML23Δ*dbpBA* (Fig 1, “pDbpBA”) restored binding to the levels of strain ML23.

The production of DbpA_VS461_, DbpA_PBr,_ or DbpA_N40-D10/E9_ in strain ML23Δ*dbpBA* resulted in decorin- and dermatan sulfate-binding significantly greater than strain ML23Δ*dbpBA* harboring vector alone, indicating that these DbpA variants provide significant adhesive function to this strain (Fig. 1). DbpA_VS461_ΔC11 conferred no detectable increase in binding, indicating, as predicted, that the 11 C-terminal amino acids of DbpA_VS461_ are essential for binding to decorin and dermatan sulfate [39]. DbpA_PBr_ promoted significantly greater spirochete binding to decorin and dermatan sulfate than did DbpA_VS461_ or DbpA_N40-D10/E9_ (Fig. 1). Thus, the degree of decorin- and dermatan sulfate-binding conferred to strain ML23Δ*dbpBA* by each DbpA variant was consistent with both the quantitative binding analysis of purified recombinant DbpA proteins described above (Fig. S2) and with our previous study of these variants expressed in a non-adherent, non-infectious strain B314 [39].

### DbpA lacking the decorin and GAG-binding activities fails to facilitate colonization

**Figure 2:**
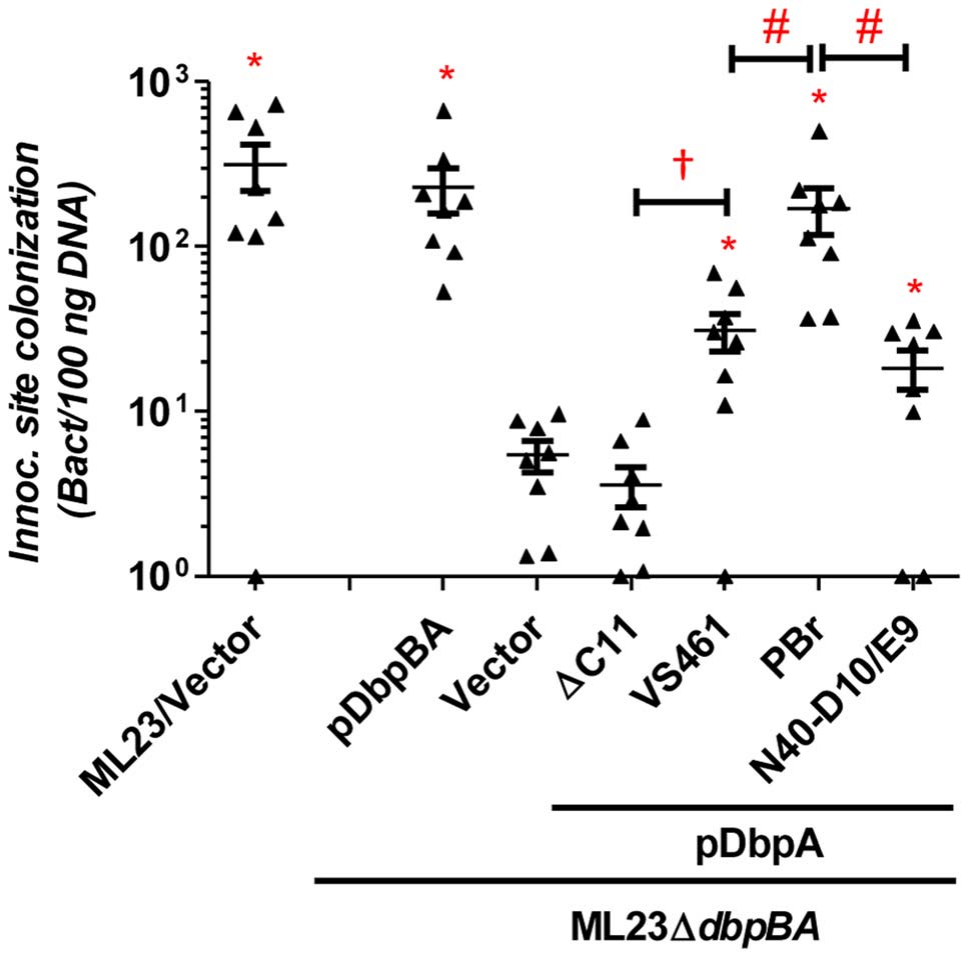
DbpA variants promote distinct *B. burgdorferi* inoculation site colonization during early infection. C3H/HeN mice infected with 10^4^ *B. burgdorferi* strain ML23/pBBE22 (“ML23/Vector”), *dbpBA* deletion strain ML23Δ*dbpBA*/pBBE22(“Vector”), or the deletion strain bearing a plasmid encoding the indicated variants were sacrificed at 3 days post-infection. Bacterial loads at the inoculation site were determined by qPCR. Data shown are the mean bacterial loads ± SEM of 10 mice per group. Statistical significance was determined using a one-way ANOVA test. Significant (P<0.05) differences in spirochete number relative to the *dbpBA* deletion strain (“*”), between two strains relative to each other (“#”), or relative to the *dbpA_VS461_ΔC11*-complemented strain (“†”) are indicated. (n.d.): not determined.

The defect in decorin- and/or dermatan sulfate-binding by DbpA_VS461_ΔC11 provided an opportunity to determine if these activities of DbpA are essential to promote *B. burgdorferi* colonization. C3H/HeN mice were infected with ML23Δ*dbpBA* producing DbpA_VS461_ or DbpA_VS461_ΔC11 and the bacterial load at the inoculation site was assessed at 3 days post-infection. Strains ML23 and ML23Δ*dbpBA*/pDbpBA were included as positive controls and colonized the site efficiently (∼300 bacteria per 100 ng of DNA), 60-fold higher than that of ML23Δ*dbpBA* harboring vector alone (Fig. 2). ML23Δ*dbpBA* producing DbpA_VS461_ promoted significant colonization (∼30 bacteria per 100 ng DNA, or ∼six-fold more than ML23Δ*dbpBA*) at the inoculation site. This finding indicated that production of DbpA alone could partially complement the defect of a *B. burgdorferi* Δ*dbpBA* mutant, consistent with previous studies [32], [33]. In contrast, ML23Δ*dbpBA* producing DbpA_VS461_ΔC11 did not mediate colonization at a level any greater than ML23Δ*dbpBA* carrying the empty vector.

**Figure 3:**
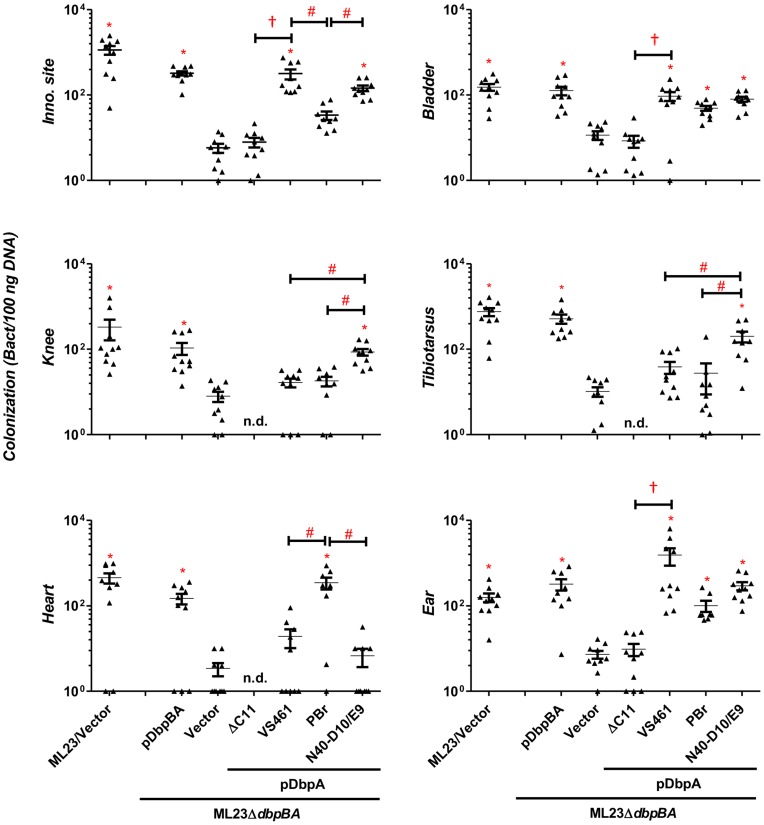
DbpA variants promote distinct *B. burgdorferi* tissue colonization profiles at 28 days post-infection. C3H/HeN mice infected with 10^4^ *B. burgdorferi* strain ML23/pBBE22 (“ML23/Vector”), *dbpBA* deletion strain ML23*ΔdbpBA*/pBBE22 (“Vector”), or the deletion strain bearing a plasmid encoding the indicated DbpA variants were sacrificed at 28 days post-infection. The bacterial loads at the inoculation site, ear, bladder, heart, knee, and tibiotarsus joint were determined by qPCR. Data shown are the mean bacterial loads ± SEM of 10 mice per group. Statistical significance was determined using a one-way ANOVA test. Significant (P<0.05) differences in spirochete number relative to the *dbpBA* deletion strain (“*”), between two strains relative to each other (“#”), or relative to the *dbpA_VS461_ΔC11*-complemented strain (“†”), are indicated. (n.d.): not determined. These data are described comprehensively with other post-infection time points in Table S1.

To determine if DbpA_VS461_ΔC11 might promote colonization at a later time point, we also assessed infected mice at 28 days post-infection. The positive control strains *B. burgdorferi* ML23 and *B. burgdorferi* ML23Δ*dbpBA*/pDbpBA displayed efficient colonization at all sites tested (Fig. 3). In particular, colonization of the inoculation site, bladder and ear was 30-200-fold higher than that of ML23Δ*dbpBA* harboring vector alone. The production of DbpA_VS461_ by ML23Δ*dbpBA* producing DbpA_VS461_ did not promote colonization of the joints or heart at this time point but did promote colonization of the inoculation site, bladder, and ear at levels indistinguishable from the positive control strains. In contrast, ML23Δ*dbpBA*/pDbpA_VS461_ΔC11 did not mediate colonization at a level any greater than ML23Δ*dbpBA* carrying the empty vector at any of the sites tested.

**Figure 4:**
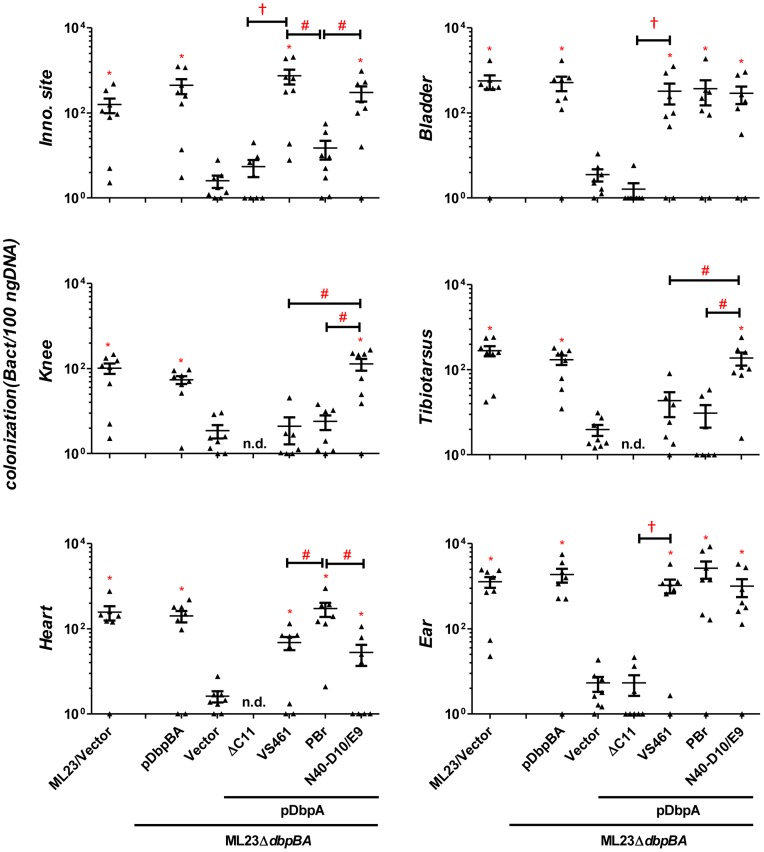
Differences in tissue tropism promoted by DbpA variants are not a function of an adaptive immune response. C3H/HeN-SCID mice infected with 10^3^ *B. burgdorferi* ML23/pBBE22 (“ML23/Vector”), ML23Δ*dbpBA*/pBBE22 (“Vector”), or ML23Δ*dbpBA* bearing a plasmid encoding the indicated DbpA variants/mutant were sacrificed at 28 days post-infection. Bacterial loads at the inoculation site, ear, bladder, heart, knee, and tibiotarsus joint were determined by qPCR. Data shown are the mean bacterial loads ± SEM of 10 mice per group. Statistical significance was determined using a one-way ANOVA test. Significant differences (P<0.05) in spirochete number relative to the ML23*ΔdbpBA* (“*”), between two strains relative to each other (“#”), or relative to the p*dbpA_VS461_ΔC11*-complemented strain (“†”), are indicated.

The bacterial load in a particular tissue may be in part a reflection of the rate of immune clearance. To determine if the colonization defect of ML23Δ*dbpBA*/pDbpA_VS461_ΔC11 might be due to the induction of a particularly robust immune response, at 28 days post-infection we measured *B. burgdorferi*-specific IgG or IgM in the sera of mice inoculated with this strain. No *B. burgdorferi*-specific antibodies were detected (Fig. S4), suggesting that ML23Δ*dbpBA*/pDbpA_VS461_ΔC11 is incapable of establishing a productive infection that triggers an adaptive immune response. In addition, the results suggest that the colonization defect of this strain was independent of an adaptive immune response. Consistent with this, a 28-day infection of the mice strain deficient for adaptive immune response (SCID mice) revealed that the production of DbpA_VS461_ΔC11 was unable to enhance the ability of ML23Δ*dbpBA* to colonize any of the tissues tested, in contrast to the production of DbpA_VS461_ (Fig. 4). Together, these results strongly suggest that the decorin- and/or dermatan sulfate-binding activity of DbpA is required for its ability to facilitate spirochetal colonization.

### Differences in the decorin- and dermatan sulfate-binding activities of DbpA variants influence colonization

**Figure 5:**
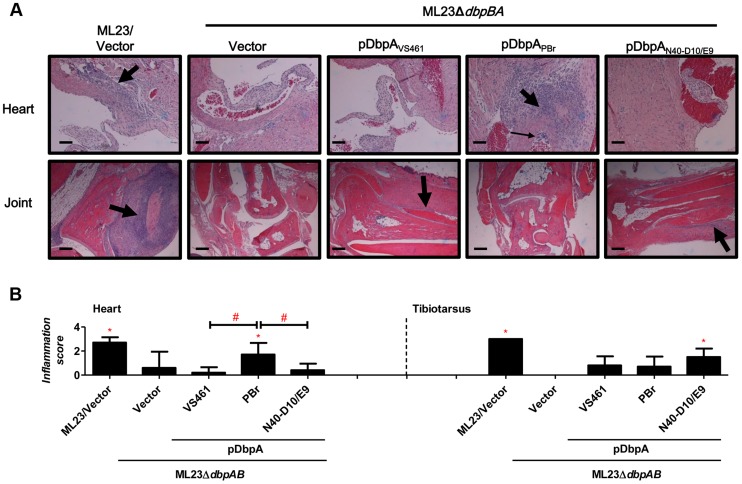
DbpA variants produced by *B. burgdorferi* lead to differences in tissue inflammation in C3H/HeN-infected mice. (A) C3H/HeN mice infected with 10^4^ *B. burgdorferi* strain ML23/pBBE22 (“ML23/Vector”), *dbpBA* deletion strain ML23Δ*dbpBA*/pBBE22 (“Vector”), or the deletion strain bearing a plasmid encoding the indicated DbpA variants were sacrificed at 28 days post-infection. Inflammation of the heart (upper panel) and joints (lower panel) were assessed using Hematoxylin and Eosin staining histopathology. The upper panel and lower panel indicate the higher-resolution (10×, bar  = 58 µm) and lower-resolution (4×, bar  = 141 µm) images. (B) To quantitate inflammation of heart and joint tissues, at least ten random sections from each infection group were scored on a scale of 0–3 for the severity of carditis and arthritis, as indicated in Material and Methods. Statistical significance was determined using a one-way ANOVA test. Data shown are the mean inflammation score ± SD of 5 mice per group. Statistical significance was determined using a one-way ANOVA test. Significant (P<0.05) differences in the inflammation score relative to the *dbpBA* deletion strain (“*”) or between two strains relative to each other (“#”), are indicated.

To test whether variation in the decorin or dermatan sulfate binding capabilities by DbpA correlates with differences in colonization and/or disease, we chose to analyze the colonization promoting abilities of three DbpA variants that display distinct decorin- and dermatan sulfate-binding activities. C3H/HeN mice were infected with ML23Δ*dbpBA* producing DbpA_PBr_, DbpA_N40-D10/E9_, or DbpA_VS461_, and differences in colonization at the inoculation site, heart, joints, bladder and ear were assessed at 3, 7, 14, 21, or 28 days post-infection. Strains ML23 and ML23Δ*dbpBA*/pDbpBA were included as positive controls, and ML23Δ*dbpBA* harboring vector alone served as a negative control. As previously observed [31], [34], [44], the kinetics of colonization by *B. burgdorferi* producing DbpA and DbpB varied with tissue: the bladder and joints were colonized by day 7 post-infection whereas the heart and ear were detectably colonized only at the 14 and 21-day time point, respectively (Figs. 3, 5 and Fig. S5; for comprehensive summary of bacterial loads at all times points, see Table S1). ML23Δ*dbpBA* harboring vector alone was defective for colonization at all time points.

Upon infection with ML23Δ*dbpBA* producing DbpA_PBr_, DbpA_N40-D10/E9_, or DbpA_VS461_, we found that at 21 days post-infection, each of the DbpA variants tested was capable of fully replacing the colonization function of the endogenous (strain B31) DbpA and DbpB in the inoculation site, bladder, knee, and tibiotarsus (Table S1). On the other hand, in the ear or heart, production of these DbpA variants was associated with delayed colonization (28- vs. 14-day colonization in the ear; 21- vs. 14-day in the heart) compared to these DbpA- and DbpB-proficient strains (Figs. 3, 5 and Fig. S5), which is consistent with the findings reported previously [44].

Importantly, in several tissues, the different DbpA variants conferred significant differences in the efficiency or kinetics of colonization. At the inoculation site at three days post-infection, production of DbpA_PBr_, which displayed greater decorin- and dermatan sulfate-binding activity than DbpA_VS461_ or DbpA_N40-D10/E9_, conferred approximately six- to nine-fold greater colonization (P<0.05; Fig. 2). The production of DbpA_PBr_ was also associated with diminished late colonization of the inoculation site, because by 28 days post-infection, ML23Δ*dbpBA* producing DbpA_PBr_ was present at this site at levels approximately 20- to 50-fold lower than ML23Δ*dbpBA* producing DbpA_VS461_ or DbpA_N40-D10/E9_ (Fig. 3).

The production of the high affinity binding variant DbpA_PBr_ also resulted in enhanced colonization of the heart at 21 and particularly 28 days post-infection compared to production of the other two DbpA variants (Figs. 3 and 5). At the later time point, ML23Δ*dbpBA* producing DbpA_PBr_ was present at levels 15- to 50-fold higher than ML23Δ*dbpBA* producing DbpA_VS461_ or DbpA_N40-D10/E9_ (P<0.05), which were not present at levels significantly higher than the negative control strain ML23Δ*dbpBA* (Fig. 3).

Interestingly, although as mentioned above, no *dbpA* allele-specific differences were observed in joint colonization at 21 days post-infection (Table S1), DbpA_N40-D10/E9_, which binds to decorin and dermatan sulfate with the lowest affinity among the variants analyzed, promoted the greatest level of colonization of the tibiotarsus and knee at 28 days post-infection (Fig. 3). Whereas by this time ML23Δ*dbpBA* producing DbpA_VS461_ or DbpA_PBr_ were no longer present in the knee or tibiotarsus at levels significantly greater than the DbpA- and DbpB-deficient ML23Δ*dbpBA* harboring vector alone, ML23Δ*dbpBA* producing DbpA_N40-D10/E9_ was present at levels 18 to 32-fold higher than strains producing either DbpA_VS461_ or DbpA_PBr_ (Fig. 3) (P<0.04).

### An adaptive immune response does not account for the differences in *B. burgdorferi* tissue tropism promoted by distinct DbpA variants

To test whether the distinct colonization levels in the inoculation site, heart, knee or tibiotarsus late in infection might be due to differences in the humoral immune response triggered by different DbpA variants, we quantitated serum IgG and IgM titers against each DbpA variant. We found no significant difference in anti-DbpA antibody production among animals infected with strains producing the different variants (Fig. S4). To determine if the ability to generate an adaptive immune response was required to elicit the differences in apparent tissue tropism among strains, we infected SCID mice with strains encoding each of the three alleles. Pilot experiments revealed that a dose of 10^3^ bacteria, i.e., ten-fold lower than that inoculated into wild type mice, was optimal for discerning colonization differences between strains ML23 and ML23Δ*dbpBA* (data not shown; see Materials and Methods). We assessed tissue burden at 28 days post-infection and found that compared to ML23Δ*dbpBA* producing DbpA_VS461_ or DbpA_N40-D10/E9_, an isogenic strain expressing DbpA_PBr_ was present at approximately five- to ten-fold lower levels at the inoculation site (P<0.02) and 15 to 87-fold higher levels in the heart (P<0.02; Fig. 4). ML23Δ*dbpBA* producing DbpA_N40-D10/E9_ colonized the knee and tibiotarsus six- to eight-fold more efficiently than ML23Δ*dbpBA* expressing DbpA_PBr_ or DbpA_VS461_ (P<0.04). Thus, the strain-specific colonization pattern for the heart and joints were identical to those observed upon infection of immunocompetent mice.

### Strain-specific DbpA variation influences carditis and arthritis

To determine whether the observed DbpA strain-specific differences in tissue tropism resulted in corresponding differences in disease severity, C3H/HeN mice infected with isogenic ML23Δ*dbpBA* derivatives producing DbpA_VS461_, DbpA_PBr_ or DbpA_N40-D10/E9_ were subjected to histopathological analysis. We evaluated carditis at the heart base at 28 days post-infection, at which time ML23Δ*dbpBA* producing DbpA_PBr_ colonized the heart at levels six to nine-fold greater than the negative control strain ML23Δ*dbpBA* or strains producing DbpA_VS461_ or DbpA_N40-D10/E9_ (Fig. 3). When coded samples were scored blindly for carditis on a scale of 0 to 3 depending on the number and intensity of inflammatory foci at the heart base (see Materials and Methods), the positive control strain ML23 induced robust (grade 3) carditis (Fig. 5B, left panel). Focal subendocardial mononuclear cell infiltrates were present upon infection by this strain (thick arrow in Fig. 5A, top row), whereas strain ML23Δ*dbpBA* harboring vector alone or producing DbpA_VS461_ or DbpA_N40-D10/E9_ showed, at most, a very mild mononuclear cell infiltrate (carditis score near grade 0; Fig. 5, left panel), reflecting their relative levels of cardiac colonization at this time point (Fig. 3). Importantly, consistent with the persistent cardiac colonization by ML23Δ*dbpBA* producing DbpA_PBr_, the mice infected with this strain exhibited significant (grade ∼2) carditis (Fig. 5B, left panel). Focal interstitial subacute myocarditis (thick arrow at Fig. 5A, top row) and vasculitis (thin arrow), involving mostly mononuclear cells was present.

Histopathological analyses were also performed on the tibiotarsus joint at 28 days post-infection, a time point at which ML23Δ*dbpBA* producing DbpA_N40-D10/E9_ colonized the joints at levels 18- to 32-fold greater than isogenic strains producing DbpA_VS461_ or DbpA_PBr_ or the negative control strain ML23Δ*dbpBA* (Fig. 3). When coded H&E-stained tibiotarsus joint samples were scored blindly for inflammatory infiltrates, severe (grade 3) arthritis was triggered by the positive control strain ML23 (Fig. 5B, right panel). Severe inflammation surrounding some tendons and of the synovial membrane was observed, with periostitis and some proliferation of new bone (thick arrow at Fig. 5A, bottom row). In contrast, the negative control *strain* ML23Δ*dbpBA* carrying vector alone or producing DbpA_PBr_ triggered no arthritis (grade 0; Fig. 5A, bottom row and Fig. 5B, right panel). The strain producing DbpA_VS461_ appeared to induce mild (grade ∼1; Fig. 5B, right) arthritis evidenced by mild and focal inflammation (thick arrow at Fig. 5A, bottom row). These findings are consistent with the low levels of joint colonization by those strains at this time point (Fig. 3). Importantly, reflecting the persistent joint colonization by ML23Δ*dbpBA* producing DbpA_N40-D10/E9_, mice infected with this strain exhibited higher levels of arthritis (grade ∼2; Fig. 5B right panel), with moderate mononuclear infiltrates commonly near connective tissue (thick arrow at Fig. 5A, bottom row). We conclude that the higher levels of heart or joint colonization associated with the production of DbpA_PBr_ or DbpA_N40-D10/E9_, respectively, resulted in greater levels of pathology.

## Discussion

Although it has long been known that different genospecies or strains of *Borrelia burgdorferi* sensu lato cause infections with different clinical manifestations in humans and distinct pathogenicity and/or tissues tropism in animal infection models, the reasons for these differences have remained obscure [9]–[12]. An attractive hypothesis put forth has been is that variation in spirochetal factors that control spread to or survival in different tissues contribute to the disparate behavior during mammalian infection [9], [12], [18], [21]. The *ospC* gene, which encodes a surface lipoprotein required for infection, is allelic variable, and a sampling of recombinant OspC variants from three invasive or noninvasive strains demonstrated a correlation between plasminogen binding and invasiveness in mice [18]. CRASP's (complement regulator acquiring surface proteins) variants, which promote serum resistance, differ in their ability to bind to the complement regulatory proteins factor H and factor H like protein (FHL-1) [16], [17], [19]. Rigorous demonstration that allelic variation of genes encoding documented or putative virulence factors influences tissue tropism and/or disease manifestation requires experimental infection using isogenic strains, and has thus far been lacking. The *dbpA* gene, which encodes a Lyme disease spirochete adhesin required for full infectivity, is allelic variable, and DbpA variants differ in their ability to promote spirochetal attachment to decorin, dermatan sulfate, or mammalian cells [21], [39]. DbpA_VS461_ΔC11, a DbpA truncation that lacks 11 C-terminal amino acids was previously shown in semi-quantitative binding assays to be unable to bind dermatan sulfate or decorin [39]. We confirmed this finding by quantitative ELISA and SPR. The C-terminal 11 amino acids lacking in DbpA_VS461_ΔC11 are not generally well conserved among DbpA variants but do encompass the universally conserved residue K170, which has been shown to be required for decorin/dermatan sulfate-binding [30], [40], [45]. To test whether the adhesive activity of DbpA is specifically required for colonization, mice were infected with a *B. burgdorferi dbpBA* deletion mutant that ectopically produced wild type DbpA_VS461_ or DbpA_VS461_ΔC11. DbpA_VS461_ΔC11 was, in fact, also unable to facilitate colonization at the inoculation site, bladder, or ear, indicating that this binding activity of DbpA is likely required for tissue colonization.

The requirement for DbpA adhesive activity for efficient mammalian colonization raised the possibility that the variability of ligand binding among DbpA variants found among Lyme disease spirochetes contributes to the observed strain-to-strain differences in tissue tropism and disease severity [21], [29], [39]. Thus, we quantitatively characterized the decorin- and dermatan sulfate-binding activities of three DbpA variants, i.e. DbpA_PBr_, DbpA_VS461_ and DbpA_N40-D10/E9_, which together represent the three major Lyme disease spirochete genospecies, and generated a set of isogenic *B. burgdorferi* strains derived from a *B. burgdorferi* Δ*dbpBA* mutant that expressed each of these variants. These DbpA-producing strains exhibited the predicted differences in their ability to bind to decorin and dermatan sulfate, with DbpA_PBr_ promoting the most efficient spirochetal binding to purified decorin and dermatan sulfate and DbpA_N40-D10/E9_ promoting the least.

When mice were infected with these strains, the *B. burgdorferi* strain producing DbpA_PBr_ promoted better early (i.e., three days post-infection) colonization at the inoculation site. This result is consistent with reports that decorin is enriched in the skin [46] and that spirochetal overproduction of DbpA enhanced colonization of the inoculation site [47]. Importantly, the strain producing DbpA_PBr_ infected the heart at levels one to two orders of magnitude greater than strains producing DbpA_VS461_ or DbpA_N40-D10/E9_, and this more intense infection of the heart was associated with enhanced carditis. *B. burgdorferi* selectively colonizes decorin-rich heart microenvironments such as the tunica adventitia [35], and upon infection, decorin-deficient mice harbor fewer *B. burgdorferi* in the heart than do littermate control mice [30]. We found that the relative tropism of the DbpA_PBr_-producing strain for skin and heart was also observed in SCID mice, indicating that the higher level of colonization of these sites by this strain is not accounted for by an adaptive immune response that might be generated more efficiently against one DbpA variant than another. Rather, the tissues that are more efficiently colonized by *B. burgdorferi* producing a variant of DbpA that binds tightly to decorin corresponds to what is currently understood about the relative enrichment of decorin in these tissues.

Nevertheless, DbpA-mediated colonization is not a simple reflection of its ability to bind decorin because DbpA_N40-D10/E9_, which displayed the weakest decorin and dermatan sulfate binding, promoted the most robust colonization of the joints late (28 days) after inoculation. Upon scoring of coded histological samples, DbpA_N40-D10/E9_ was the only variant that promoted arthritis significantly more severe than the non-DbpA-producing control strain. Our *in vitro* assays indicate that compared to DbpA_VS461_ or DbpA_PBr_, DbpA_N40-D10/E9_ poorly recognizes human recombinant decorin and the (commercially available porcine skin) dermatan sulfate utilized in this study. However, it is possible that DbpA_N40-D10/E9_ binds to a host ligand present in the murine joint better than these other DbpA variants. Dermatan sulfate, like other GAGs, is heterogeneous with respect to epimerization and modification, raising the possibility that murine joint decorin may be well recognized by DbpA_N40-D10/E9_. In addition, biglycan, which like the other class I proteoglycan decorin contains ten leucine-rich repeats and two dermatan sulfate GAGs, is present in the joint at higher levels than decorin and could be an additional (well recognized) ligand for DbpA_N40-D10/E9_ [48]–[50]. Finally, the tibiotarsus joint apparently presents *B. burgdorferi* with functionally distinct microenvironments, because *B. burgdorferi* colonizes both synovial and adjacent connective tissues in the joints of untreated SCID mice, but are cleared specifically from synovium by administration of anti-DbpA serum [51]. In our study, the tropism of DbpA_N40-D10/E9_ for joints was not a simple function of adaptive immunity because it was recapitulated in SCID mice, but it is possible that the different DbpA variants, by recognizing host ligands differently, promote distinct distributions of spirochetes among joint microenvironments. A technical challenge to experimental validation of this hypothesis is the relative paucity of spirochetes in the joints of infected animals.

One interesting observation is that an allele of *dbpA* from a *B. burgdorferi* sensu stricto strain (i.e. strain N40-D10/E9) promoted joint colonization and disease in the mouse, an apparent tropism that correlates with the common manifestation of Lyme arthritis upon infection by this genospecies of Lyme disease spirochete [10]. This is not to imply, however, that the production of a particular DbpA variant fully explains the tissue tropism of a given strain. Tissue tropism is undoubtedly multifactorial, so any approach that addresses the contribution of a single allelic variable gene, in this case *dbpA*, is inherently limited due to its concomitant inability to assess the role of other potential determinants. In addition, here we assessed strains that did not produce DbpB, which may have partially redundant function, and to what degree allelic variation of *dbpA* contributes to the etiology of distinct symptoms associated with different Lyme disease strains in otherwise wild-type strains will require further study. Nevertheless, the demonstration that *dbpA* influences colonization and disease by the Lyme disease spirochete in an allele-specific manner provides important support for the long-postulated model that allelic variation of a *Borrelia* surface protein influences tissue tropism.

## Materials and Methods

### Ethics statement

All mouse experiments were performed in strict accordance with all provisions of the Animal Welfare Act, the Guide for the Care and Use of Laboratory Animals, and the PHS Policy on Humane Care and Use of Laboratory Animals. The protocol was approved by the Tufts University School of Medicine Institutional Animal Care and Use Committee (IACUC), protocol docket number 2011–140. All efforts were made to minimize animal suffering.

### Bacterial strains and growth conditions

The *Borrelia* and *E. coli* strains used in this study are described in Table S3. *Escherichia coli* strains DH5α, BL21 and derivatives were grown in Luria-Bertani (BD Bioscience, Franklin lakes, NJ) broth or agar, supplemented with kanamycin (50 µg/ml) or ampicillin (100 µg/ml) where appropriate. All *B. burgdorferi* strains were grown in BSK-II completed medium supplemented with kanamycin (200 µg/ml) or Gentamycin (50 µg/ml).

### Generation of recombinant DbpA proteins and antisera

To generate recombinant histidine-tagged DbpA proteins, the *dbpA* open reading frames lacking the putative signal sequences from *B. burgdorferi* strains B31 and N40-D10/E9, *B. garinii* strain PBr, and *B. afzelii* strain VS461 were inserted into pET15b (Novagen, Madison, WI) as previously described [39] (see Table S3). In addition, *dbpA* open reading frames (lacking the putative signal sequence) from *B. burgdorferi* strain 297 (encoding residues 30 to 187), B356 (encoding residues 33 to 194), and an altered open reading frame encoding DbpA_VS461_ΔC11 (residues 22 to 158, lacking the 11 C-terminal amino acids, from *B. afzelii* strain VS461), were amplified using the primers described in Table S3. Amplified fragments were engineered to encode a *BamH*I site at the 5′ end and a stop codon followed by a *Sal*I site at the 3′ end. PCR products were sequentially digested with *BamH*I and *Sal*I and then inserted into the *BamH*I and *Sal*I sites of pQE30 (Qiagen, Valencia, CA). The resulting plasmids were transformed into *E. coli* strain M15 (for *dbpA_297_, dbpA_B356,_* and *dbpA_VS461_ΔC11*) or BL21 (for all other *dbpA* alleles) and the plasmid inserts were sequenced (Tufts core sequencing facility). The histidine-tagged DbpA variants were produced and purified by nickel affinity chromatography according to the manufacturer's instructions (Qiagen, Valencia, CA). Antisera against DbpA_N40-D10/E9_, DbpA_PBr_, or DbpA_VS461_ were generated by immunizing five-week-old BALB/C mice with each of the DbpA proteins as described previously [51].

### Purification of human decorin

Recombinant human decorin, a generous gift from David Mann (MedImmune, Inc.), was purified from stably transfected Chinese hamster ovary cells (ATCC CCL 61) as described previously [52].

### Circular dichroism (CD) spectroscopy

CD analysis was performed on a Jasco 810 spectropolarimeter (Jasco Analytical Instrument, Easton, MD) under N_2_. CD spectra were measured at RT (25°C) in a 1 mm path length quartz cell. Spectra of DbpA_VS461_ (10 µM) and DbpA_VS461_ΔC11 (10 µM) were recorded in Tris buffer at 25°C, and three far-UV CD spectra were recorded from 190 to 250 nm for far-UV CD in 1 nm increments. The background spectrum of buffer without protein was subtracted from the protein spectra. CD spectra were initially analyzed by the software Spectra Manager Program. Analysis of spectra to extrapolate secondary structures was performed by Dichroweb (http://dichroweb.cryst.bbk.ac.uk/html/home.shtml) using the K2D and Selcon 3 analysis programs [53].

### GAG and decorin binding assays

Quantitative ELISA for decorin and dermatan sulfate binding by DbpA proteins was performed similarly to that previously described [54]. One µg of decorin, dermatan sulfate, chondroitin 6 sulfate, or BSA was coated onto microtiter plate wells. One hundred microliters of increasing concentrations (0.03125, 0.0625, 0.125, 0.25, 0.5, 1, 2 µM) of histidine-tagged RevA (negative control) or a DbpA variant, including DbpA_B31_, DbpA_297_, DbpA_N40-D10/E9_, DbpA_B356_, DbpA_VS461_, DbpA_PBr_, or DbpA_VS461_ΔC11, were then added to the wells. To detect the binding of histidine-tagged proteins, mouse anti-histidine tag (Sigma-Aldrich, St. Louis, MO; 1∶200) and HRP-conjugated goat anti-mouse IgG (Promega, Fitchburg, WI; 1∶1,000) were used as primary and secondary antibodies. The plates were washed three times with PBST (0.05% Tween20 in PBS buffer), and 100 µl of tetramethyl benzidine (TMB) solution (Kirkegaard and Perry Laboratories, Gaithersburg, MD) were added to each well and incubated for five minutes. The reaction was stopped by adding 100 µl of 0.5% hydro sulfuric acid to each well. Plates were read at 405 nm using a Synergy HT ELISA plate reader (BioTek, Winooski, VT). To determine the dissociation constant (K_D_), the data were fitted by the following equation using KaleidaGraph software (Version 4.1.1 Abekbecj Software, Reading, PA).
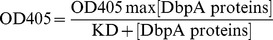
(1)


### Surface Plasmon Resonance (SPR)

Interactions of DbpA with decorin or dermatan sulfate were analyzed by a SPR technique using a Biacore 3000 (GE Healthcare, Piscataway, NJ). Ten µg of biotinylated decorin or dermatan sulfate was conjugated to an SA chip (GE Healthcare, Piscataway, NJ). A control flow cell was injected with PBS buffer without decorin or dermatan sulfate. For quantitative SPR experiments to determine decorin- or dermatan sulfate–binding, ten µl of increasing concentrations (0, 15.625, 31.25, 62.5, 125, 250, 500 nM) of a DbpA variant, including DbpA_B31_, DbpA_297_, DbpA_N40-D10/E9_, DbpA_B356_, DbpA_VS461_, DbpA_PBr_, or DbpA_VS461_ΔC11, were injected into the control cell and flow cell immobilized with decorin or dermatan sulfate at 10 µL/min, 25°C. To obtain the kinetic parameters of the interaction, sensogram data were fitted by means of BIAevaluation software version 3.0 (GE Healthcare, Piscataway, NJ), using the one step biomolecular association reaction model (1∶1 Langmuir model), resulting in optimum mathematical fit with the lowest Chi values.

### Shuttle plasmid construction

To generate the plasmids encoding *dbpA* alleles, genes *dbpA_N40-D10/E9_*, *dbpA_VS461_*, *dbpA_PBr_*, or *dbpA_VS461_ΔC11* were first PCR amplified with the addition of a *Sal*I site and a *BamH*1 site at the 5′ and 3′ ends, respectively, using the primers listed in Table S1. Amplified DNA fragments were inserted into TA cloning vector pCR2.1-TOPO (Invitrogen, Houston, TX; see Table S3), to generate the plasmids pCR2.1- *dbpA_N40-D10/E9_*, pCR2.1-*dbpA_VS461_*, pCR2.1-*dbpA_PBr_*, and pCR2.1-*dbpA_VS461_ΔC11*. The plasmids were then digested with *Sal*I and *BamH*I to release the *dbpA* alleles, which were then inserted into the *Sal*I and *BamH*I sites of pBBE22 (see Table S3). The promoter region of *dbpBA* from *B. burgdorferi* B31, 289 bp upstream from the start codon of *dbpB*, was also PCR amplified, adding, *Sph*I and *Sal*I sites at the 5′ and 3′ ends, respectively, using primers pdbpBAfp and pdbpBArp (Table S3). Promoter fragments were then inserted into the *Sph*I and *Sal*I sites of pBBE22 to drive the expression of *dbpA_N40-D10/E9_*, *dbpA_VS461_*, *dbpA_PBr_*, and *dbpA_VS461_ΔC11*.

### Plasmid transformation into *B. burgdorferi*

Electrocompetent *B. burgdorferi* ML23Δ*dbpBA* was transformed separately with 80 µg of each of the shuttle plasmids encoding *dbpA_N40-D10/E9_, dbpA_VS461_, dbpA_PBr_, or dbpA_VS461_ΔC11* (see Table S3) and cultured in BSK II medium at 33°C for 24 hours. Aliquots of the culture were mixed with 1.8% analytical grade agarose (BioRad; Hercules, CA) and plated on a solidified BSK II/agarose layer in sterilized 100×20 mm tissue culture dishes (Corning Incorporated, Corning, NY). Plates were incubated at 33°C in 5% CO_2_ for two weeks. Kanamycin- and gentamycin-resistant colonies of *dbpA*-complemented *B. burgdorferi* were obtained and expanded at 33°C in liquid BSK II medium containing kanamycin and gentamycin, followed by genomic DNA preparation as previously described [55]. PCR was performed with primers (Fig. S1) specific for *kan* (encoding the kanamycin resistance gene), to verify its presence in the transformants. The plasmid profiles of the *dbpBA* deficient mutant complemented with *dbpA* alleles were examined as described previously [56] and found to be identical to those of this strain harboring the empty vector (data not shown).

### Flow cytometry

To determine the production and the surface localization of DbpA variants and of OspC in *B. burgdorferi*, 1×10^8^ *B. burgdorferi* cells were washed thrice with HBSC buffer containing DB (25 mM Hepes acid, 150 mM sodium chloride, 1 mM MnCl_2_, 1 mM MgCl_2_, 0.25 mM CaCl_2_, 0.1% glucose, and 0.2% BSA, final concentration) and then resuspended into 500 µL of the same buffer. A mixture of mouse antisera raised against DbpA_B31_, DbpA_N40-D10/E9_, DbpA_VS461_, and DbpA_PBr_ [39] and rabbit anti-OspC (Rockland, Gilbertsville, PA) was used as a primary antibody, and Alexa488-conjugated goat anti-mouse IgG (Invitrogen; 1∶250×) and Alexa 635-conjugated goat anti-rabbit IgG (Invitrogen; 1∶250×) were used as secondary antibodies. 300 µL of formalin (0.1%) was then added for fixing. Surface production of DbpA and OspC was measured by flow cytometry using a Becton-Dickinson FACSCalibur (BD Bioscience, Franklin Lakes, NJ). All flow cytometry experiments were performed within two days of collection of *B. burgdorferi* samples. Spirochetes in the suspension were distinguished on the basis of their distinct light scattering properties in a Becton Dickinson FACSCalibur flow cytometer equipped with a 15 mW, 488 nm air-cooled argon laser, a standard three-color filter arrangement, and CELLQuest Software (BD Bioscience, Franklin Lakes, NJ). The mean fluorescence index (MFI) of each sample was obtained from FlowJo software (Three star Inc, Ashland, OR) representing the surface production of the indicated proteins. To compare the surface production of DbpA and OspC proteins in different strains, results in Fig. S3 are shown as relative production, the MFI normalized to that of *B. burgdorferi* strain ML23. The results shown in Fig. S3 represent the mean of twelve independent determinations ± the standard deviation. Each standard deviation value was no more than 7 percent of its mean value.

### Binding of radiolabeled *B. burgdorferi* to purified decorin or GAG

Binding of *B. burgdorferi* to purified decorin or dermatan sulfate was determined as previously described [39]. Briefly, spirochetes were radiolabeled with [^35^S] methionine, and 1×10^8^ radiolabeled bacteria were added to break-apart microtiter plate wells previously incubated with 250 µg/mL decorin, dermatan sulfate or chondroitin 6 sulfate (as a negative control). After 16 hours at 4°C, unbound bacteria were removed by washing with PBS containing 0.2% BSA. Plates were air-dried, and percent binding was determined by liquid scintillation counting. The percentage of bound bacteria was determined by radioactive counts in bound bacteria normalized to the counts in the inoculum.

### Mouse infection experiments

Four-week-old female C3H/HeN mice (Charles River, Wilmington, MA) were used for all experiments. Mice were infected by intradermal injection as previously described [31] with ∼10^4^ *B. burgdorferi* ML23Δ*dbpBA*/vector, or derivatives expressing *dbpA_N40-D10/E9_*, *dbpA_VS461_*, *dbpA_PBr_*, or *dbpA_VS461_ΔC11*. For the mice sacrificed at 3 days post-infection, the skin at the inoculation site was collected. For the mice sacrificed at 7, 14, 21, or 28 days post-infection, skin at the inoculation site, the tibiotarsal joint, knee joint, bladder, heart, and ear were collected. For infections of mice defective in adaptive immunity, four-week-old C3H-SCID mice (Jackson Lab, Bar Harbor, ME) were infected as described above for C3H/HeN mice. In C3H-SCID mice, a dose of 10^3^ resulted in a 30- to 236-fold difference in bacterial load of *B. burgdorferi* strain ML23 and ML23Δ*dbpBA*/vector, whereas a dose of 10^4^ resulted in indistinguishable colonization by two strains. Hence to maximize the chances of revealing differences in colonization due to the production of the DbpA variants, the lower (i.e. 10^3^) dose was used in this study. All SCID mice were sacrificed on 28 days post-infection, and skin at the inoculation site, the tibiotarsal joint, knee joint, bladder, heart, and ear were collected.

### Quantification of *B. burgdorferi* in infected tissues and blood samples

DNA was extracted from tissue using the DNeasy Blood & Tissue kit (Qiagen). The quantity and quality of DNA for each tissue sample have been assessed by measuring the concentration of DNA and the ratio of the UV absorption at 280 to 260. The amount of DNA used in this study was 100 ng for each sample, and the 280∶260 ratio was between 1.75 to 1.85, indicating the lack of contaminating RNA or proteins. qPCR was then performed to quantitate bacterial load, using 100 ng of DNA per reaction. *B. burgdorferi* genomic equivalents were calculated using an CFX Connect Real-Time PCR detection system (BioRad, Hercules, CA) in conjunction with SYBR green PCR Mastermix (BioRad), based on amplification of the *B. burgdorferi recA* gene using primers BBRecAfp and BBRecArp (Table S2), as described previously [57]. The number of *recA* copies was calculated by establishing a threshold cycle (Ct) standard curve of a known number of *recA* gene extracted from *B. burgdorferi* strain B31, then comparing the Ct values of the experimental samples. To assure the low signals were not simply a function of the presence of PCR inhibitors in the DNA preparation, we subjected 5 samples from tibiotarsal joint, bladder, and heart of the mice infected by *B. burgdorferi* strain ML23/vector, ML23Δ*dbpBA*/vector (i.e. the *dbpBA* mutant), or *dbpBA* mutant complemented with *dbpA_N40_, dbpA_VS461_, dbpA_PBr_*, or *dbpA_VS461_ΔC11* to qPCR using mouse nidogen primers mNidfp and mNidrp (Table S3) as an internal standard [58]. As predicted, we detected 10^7^ copies of the nidogen gene from 100 ng of each DNA sample, ruling out the presence of PCR inhibitors in these samples.

### Antibody titer determinations

Nunc maxisorp flat-bottom 96-well plates were coated with 1 µg of recombinant DbpA_B31_, DbpA_N40-D10/E9_, DbpA_PBr_, or DbpA_VS461_ protein in 100 µl of coating buffer (0.05 M Na_2_CO_3_, pH 9.0) overnight in 4°C. The next day, plates were washed three times with wash buffer (0.05% PBS Tween 20) and blocked for 1 hour in blocking buffer (0.05% PBS Tween 20 with 1% BSA). Plates were then washed three times, and incubated for 1 hour with serum (diluted 1∶100, 1∶300 and 1∶900) at room temperature. Then, after washing plates three times, a 1∶10,000 dilution of HRP-conjugated goat anti-mouse IgM or IgG antibodies (Bethyl Lab, Montgomery, TX) was added to each well for one hour at room temperature. Subsequently, plates were washed and 100 µl of SureBlue Reserve TMB 2-Component Microwell Peroxidase Substrate system (Kirkegaard and Perry Laboratories) were added to each well. Plates were then read at OD_650_ using a Synergy HT ELISA plate reader (BioTek). For kinetic ELISA experiments, readings were taken every minute for 10 minutes. V_max_ (milli-optical density unit per minute) based on the slope of the continuous readings were calculated using the Gen5 Software (Version 2.00.18, BioTek, Winooski, VT).

Controls included three dilutions (1∶100, 1∶300 and 1∶900) of purified IgG or IgM (125 µg/mL; Bethyl Lab) coated on microtiter plates, and uninfected (“naïve”) serum run in parallel with sample sera. The product of V_max_ × inverse serum dilution factor was largely independent of serum dilution factor. Arbitrary units of a given serum sample were chosen as the largest V_max_ × inverse serum dilution factor product within the dilution range, and were expressed relative to the arbitrary units of control pooled sera, set to 100 (Marty-Roix, R. and Maung, N., unpublished data). Antibody units of sample sera were normalized by subtracting the antibody unit “background” of naïve mice, and expressed relative to the control wells coated with purified IgG and IgM.

### Histological evaluation of arthritis and carditis

At least 10 tibiotarsus joints and 5 hearts were collected from each group of mice (5 animal/group) infected with the different *B. burgdorferei* isolates. For histology, joints and hearts were fixed in 10% formalin and processed for Hematoxylin and Eosin staining. Sections were evaluated for signs of arthritis using histological parameters for *B. burgdorferi*-induced inflammation [51], [59], such as exudation of inflammatory cells into joints, altered thickness of tendons or ligament sheaths, and hypertrophy of the synovium. Signs of carditis [51], [60] were evaluated based on cardiac inflammatory infiltrate, including transmural infiltration of neutrophils in the blood vessels and infiltration by macrophages into the surrounding connective tissue. Inflammation was scored as 0 (no inflammation), 1 (mild inflammation with less than two small foci of infiltration), 2 (moderate inflammation with two or more foci of infiltration), or 3 (severe inflammation with focal and diffuse infiltration covering a large area).

### Statistical analysis

Significant differences between samples were determined using the one-way ANOVA test following logarithmic transformation of the data. P-values were determined for each sample.

## Supporting Information

Figure S1 **The 11 C-terminal amino acids of DbpA_VS461_ do not affect its structure.** Far-UV CD analysis of DbpA_VS461_ and DbpA_VS461_ΔC11. The molar ellipticity, Φ, was measured from 190 to 250 nm for 10 µM of each protein in PBS buffer.
(TIF)

Figure S2 **Recombinant DbpA variants exhibit distinct decorin- and dermatan sulfate-binding activities. Left panel**: The indicated concentrations of various recombinant histidine-tagged DbpA variants, including DbpA_B31_ (“B31”), DbpA_N40-D10/E9_ (“N40-D10/E9”), DbpA_297_ (“297”), DbpA_B356_ (“B356”), DbpA_PBr_ (“PBr), DbpA_VS461_ (“VS461”), DbpA_VS461_ΔC11 (“VS461ΔC11”), or RevA (negative control), were added to quadruplicate wells coated with (**A**) decorin or (**B**) dermatan sulfate (Derm SO_4_)., and protein binding was quantitated by ELISA (Y axis). Numbers represent the mean ± standard deviation. Binding of DbpA_VS461_ΔC11 to decorin and dermatan sulfate was not statistically different than RevA (p>0.05 by Student's t test). (The other DbpA variants bound to these substrates significantly better than RevA, and their *K_D_* was obtained from the average of three independent experiments is shown on Table 1. Shown is a representative of three independently performed experiments. **Right panel**: 15.625 to 500 nM of histidine-tagged DbpA protein was flowed over a surface coated with 10 µg (**A**) decorin or (**B**) dermatan sulfate (Derm SO_4_). Binding was measured in response units (RU) by SPR (see Materials and Methods). Shown is a representative of six experiments performed on three different occasions and in Table 1 are the *k_on_, k_off_,* and *K_D_* values obtained from average of these six experiments.
(TIF)

Figure S3 **DbpA variants are localized at the surface of** ***B. burgdorferi*** **.** Flow cytometry analysis of DbpA localized on the surface of *B. burgdorferi*. (**A**) Flow cytometry analysis of DbpA localized to the surface of parental strain *B. burgdorferi* ML23/pBBE22 (“ML23/Vector”), *dbpBA* deletion strain ML23*ΔdbpBA*/pBBE22 (“ML23Δ*dbpBA*/Vector”), and the *dbpBA* deletion strain bearing a plasmid encoding DbpBA (“ML23*ΔdbpBA*/pDbpBA”). (**B**) The production of OspC (control) and DbpA on the surface of *B. burgdorferi* strain ML23/pBBE22 (“ML23/vector”), *dbpBA* deletion strain ML23*ΔdbpBA*/pBBE22 (“Vector”), and the deletion strain bearing a plasmid encoding the indicated DbpA variants were detected by flow cytometry (see Materials and Methods). Non-adherent *B. burgdorferi* strain B314 carrying the shuttle vector (“B314/Vector”) was included as a negative control. Values are shown relative to the production levels of DbpA on the surface of *B. burgdorferi* strain ML23/pBBE22 (“ML23/vector”). Each bar represents the mean of twelve independent determinations ± the standard deviation. Each standard deviation value is no more than 7 percent of its mean value. (*): indicates that surface production of the indicated proteins was significantly lower (P<0.05) than surface production of DbpA by *B. burgdorferi* strain ML23/pBBE22.
(TIF)

Figure S4 **DbpA variants produced in** ***B. burgdorferi*** **trigger similar adaptive immune responses.** C3H/HeN mice infected with *B. burgdorferi* strain ML23/pBBE22 (“ML23/Vector”), *dbpBA* deletion strain ML23Δ*dbpBA*/pBBE22 (“Vector”), or the deletion strain bearing a plasmid encoding DbpA (“pDbpBA”), DbpA_VS461_ (“VS461”), DbpA_PBr_ (“PBr”), DbpA_N40-D10/E9_ (“N40-D10/E9”), or DbpA_VS461_ΔC11 (“ΔC11”) at doses of 10^4^ spirochetes, were sacrificed at 28 days post-infection. Serum titers of IgG (top panel) and IgM (bottom panel) in mice infected with the indicated strains. Statistical significance was determined using a one-way ANOVA test. Data shown are the mean bacterial loads ± SEM of 8 mice per group. Statistical significance was determined using a one-way ANOVA test. Significant (P<0.05) differences in antibody titers relative to the *dbpBA* deletion strain (“*”) or relative to the *dbpA_VS461_ΔC11*-complemented strain (“†”), are indicated.
(TIF)

Figure S5 **The kinetics of** ***B. burgdorferi*** **dissemination in C3H/HeN mice.** C3H/HeN mice infected (10^4^ spirochetes) with *B. burgdorferi* strain ML23/pBBE22 (“ML23/Vector”), *dbpBA* deletion strain ML23*ΔdbpBA*/pBBE22 (“Vector”), or the deletion strain bearing a plasmid encoding the indicated DbpA variants were sacrificed at 7 and 14 days post-infection. Bacterial loads at the (**A**) inoculation site, (**B**) knee joint, (**C**) heart, (**D**) bladder, (**E**) tibiotarsus joint, and (**F**) earwere determined by qPCR. Data shown are the mean bacterial loads ± SEM of 10 mice per group. Statistical significance was determined using a one-way ANOVA test. Significant (P<0.05) differences in spirochete number relative to the *dbpBA* deletion strain (“*”), or between two strains relative to each other (“#”) are indicated. These data are described comprehensively with other post-infection time points in Table S1.
(TIF)

Figure S6 **Sequence alignment of DbpA variants found in B31, 297, N40-D10/E9, PBr and VS461 strains for** ***Borrelia*** **.** DbpAB356 is not shown because it has 99% sequence identity with strain DbpA_N40-D10/E9_ [39]. Black shaded residues are the critical residues for decorin- and dermatan sulfate-binding [41]. Gray shaded residues are the residues in BXBB motif previously suggested to be important for decorin- and dermatan sulfate-binding [40], [45].
(TIF)

Table S1 **Summary of tissue colonization promoted by diverse** ***dbpA*** **alleles.**
(DOCX)

Table S2 **Primers used in this study.**
(DOCX)

Table S3 **Bacterial strains and plasmids used in this study.**
(DOCX)

Text S1 **Supplemental references.**
(DOCX)
