# Supplementary figures and images for: Strain-Specific Variation of the Decorin-Binding Adhesin DbpA Influences the Tissue Tropism of the Lyme Disease Spirochete
Source: PLoS Pathog. 2014 Jul 31;10(7):e1004238. doi: 10.1371/journal.ppat.1004238 (PMC4117581; doi:10.1371/journal.ppat.1004238)

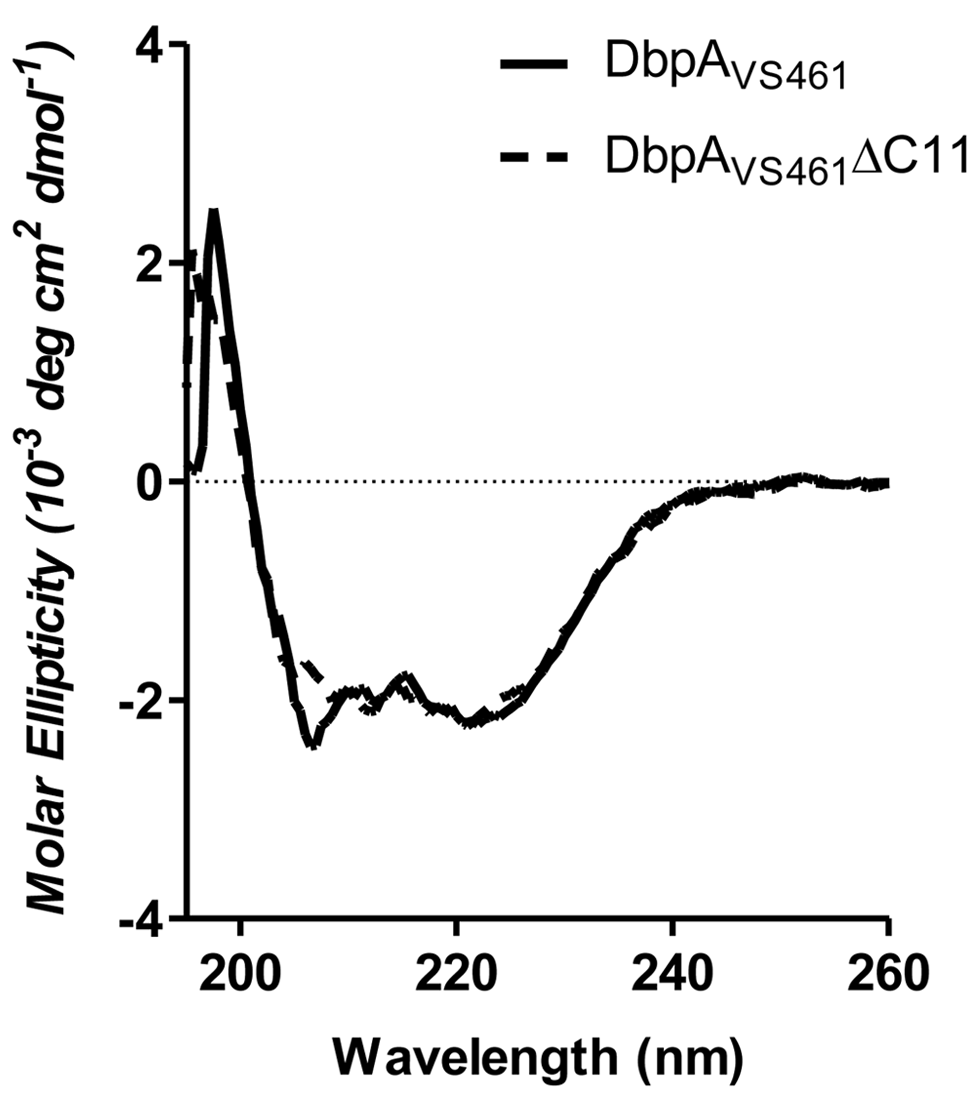

Supplement: Figure S1 — The 11 C-terminal amino acids of DbpAVS461 do not affect its structure. Far-UV CD analysis of DbpAVS461 and DbpAVS461ΔC11. The molar ellipticity, Φ, was measured from 190 to 250 nm for 10 µM of each protein in PBS buffer. (TIF) [file ppat.1004238.s001.tif]

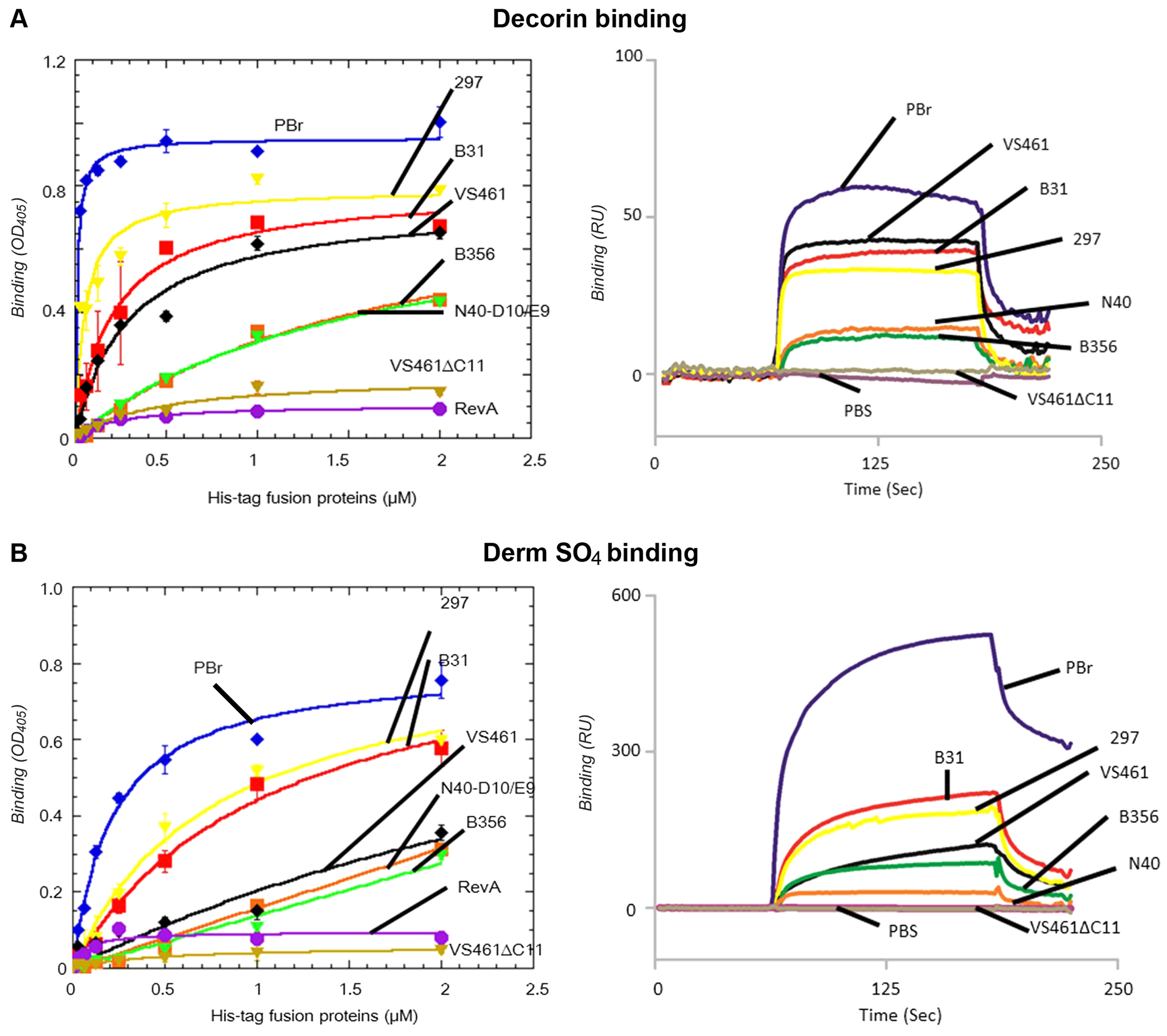

Supplement: Figure S2 — Recombinant DbpA variants exhibit distinct decorin- and dermatan sulfate-binding activities. Left panel: The indicated concentrations of various recombinant histidine-tagged DbpA variants, including DbpAB31 (“B31”), DbpAN40-D10/E9 (“N40-D10/E9”), DbpA297 (“297”), DbpAB356 (“B356”), DbpAPBr (“PBr), DbpAVS461 (“VS461”), DbpAVS461ΔC11 (“VS461ΔC11”), or RevA (negative control), were added to quadruplicate wells coated with (A) decorin or (B) dermatan sulfate (Derm SO4)., and protein binding was quantitated by ELISA (Y axis). Numbers represent the mean ± standard deviation. Binding of DbpAVS461ΔC11 to decorin and dermatan sulfate was not statistically different than RevA (p>0.05 by Student's t test). (The other DbpA variants bound to these substrates significantly better than RevA, and their KD was obtained from the average of three independent experiments is shown on Table 1. Shown is a representative of three independently performed experiments. Right panel: 15.625 to 500 nM of histidine-tagged DbpA protein was flowed over a surface coated with 10 µg (A) decorin or (B) dermatan sulfate (Derm SO4). Binding was measured in response units (RU) by SPR (see Materials and Methods). Shown is a representative of six experiments performed on three different occasions and in Table 1 are the kon, koff, and KD values obtained from average of these six experiments. (TIF) [file ppat.1004238.s002.tif]

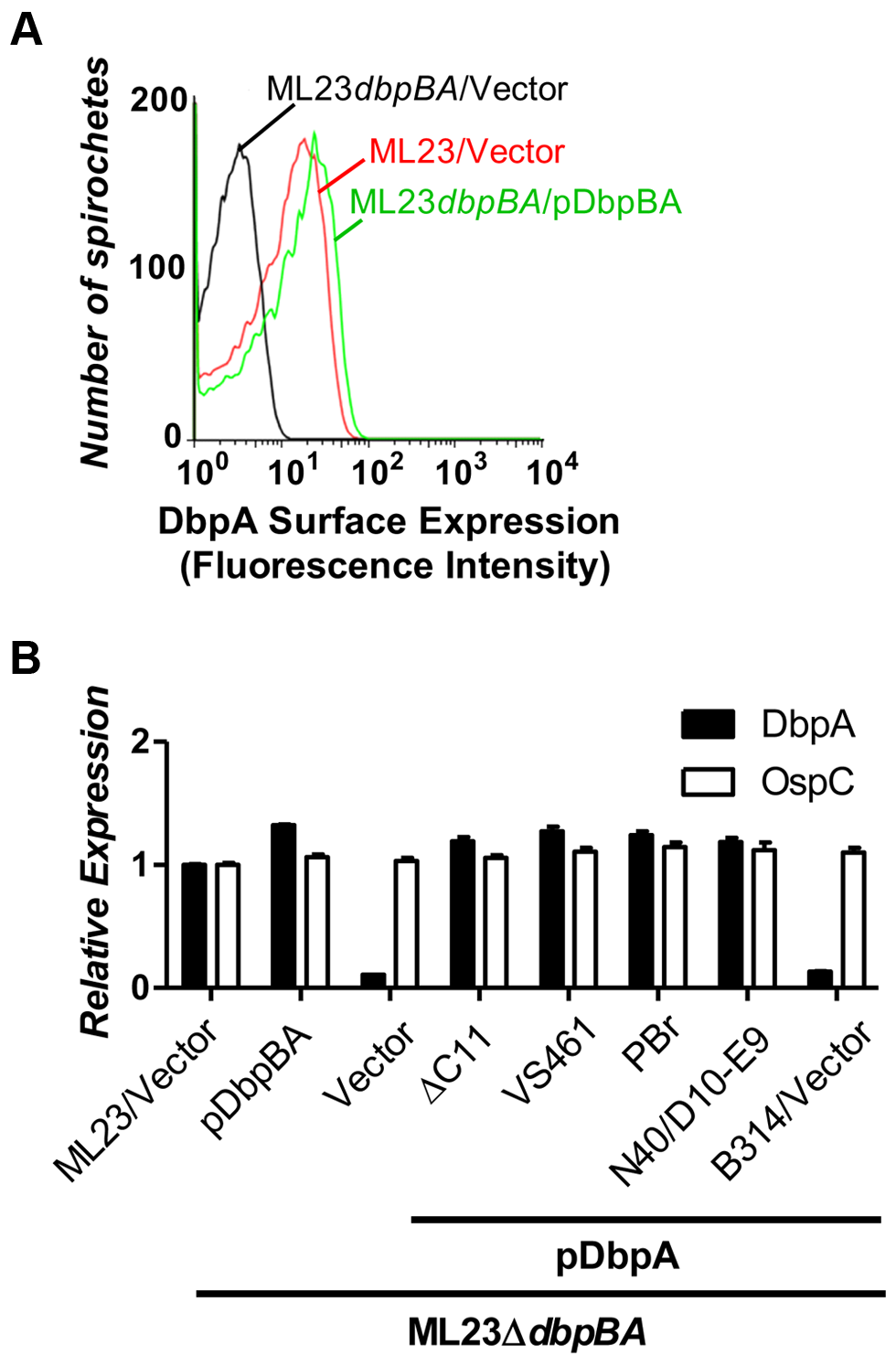

Supplement: Figure S3 — DbpA variants are localized at the surface of B. burgdorferi . Flow cytometry analysis of DbpA localized on the surface of B. burgdorferi. (A) Flow cytometry analysis of DbpA localized to the surface of parental strain B. burgdorferi ML23/pBBE22 (“ML23/Vector”), dbpBA deletion strain ML23ΔdbpBA/pBBE22 (“ML23ΔdbpBA/Vector”), and the dbpBA deletion strain bearing a plasmid encoding DbpBA (“ML23ΔdbpBA/pDbpBA”). (B) The production of OspC (control) and DbpA on the surface of B. burgdorferi strain ML23/pBBE22 (“ML23/vector”), dbpBA deletion strain ML23ΔdbpBA/pBBE22 (“Vector”), and the deletion strain bearing a plasmid encoding the indicated DbpA variants were detected by flow cytometry (see Materials and Methods). Non-adherent B. burgdorferi strain B314 carrying the shuttle vector (“B314/Vector”) was included as a negative control. Values are shown relative to the production levels of DbpA on the surface of B. burgdorferi strain ML23/pBBE22 (“ML23/vector”). Each bar represents the mean of twelve independent determinations ± the standard deviation. Each standard deviation value is no more than 7 percent of its mean value. (*): indicates that surface production of the indicated proteins was significantly lower (P<0.05) than surface production of DbpA by B. burgdorferi strain ML23/pBBE22. (TIF) [file ppat.1004238.s003.tif]

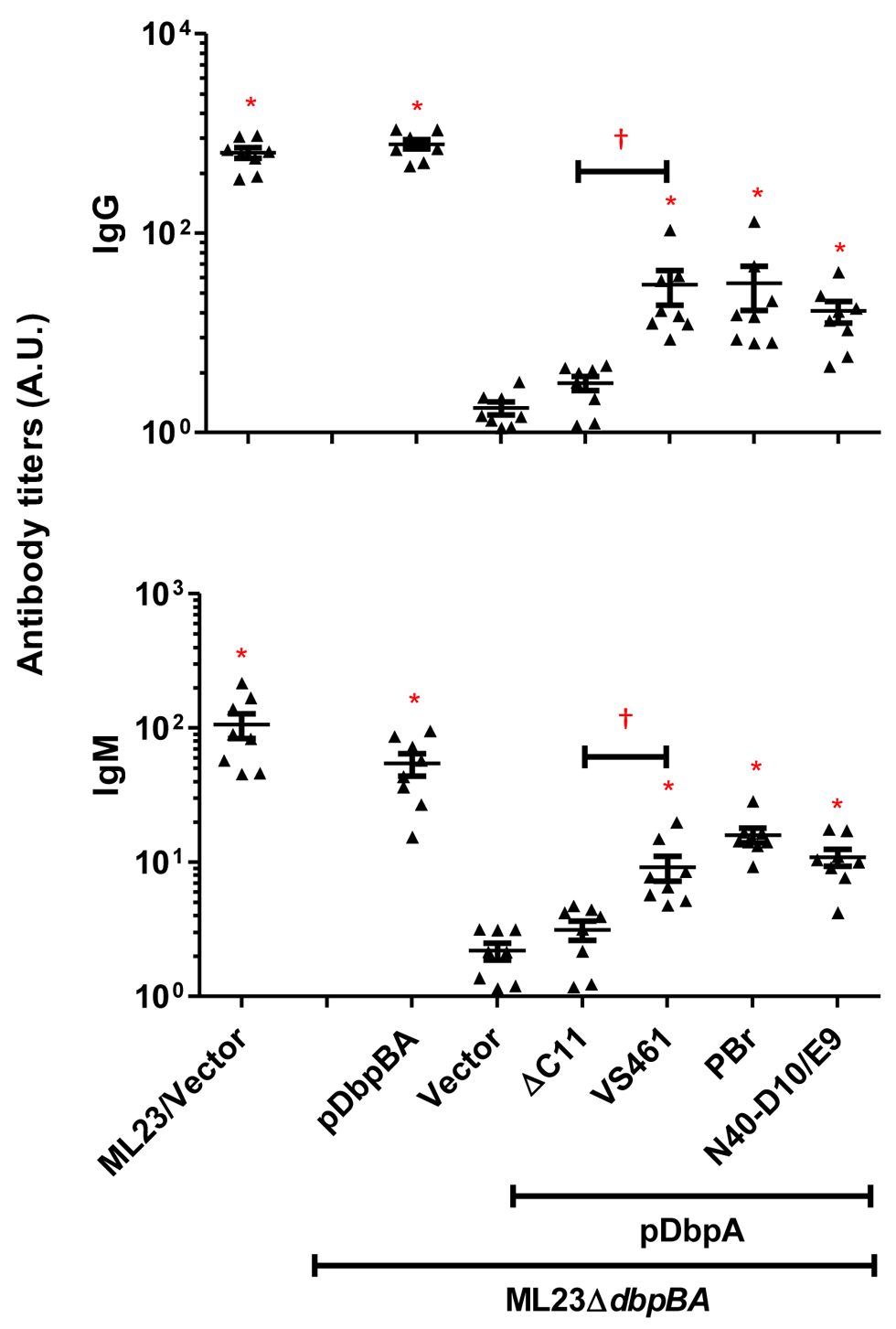

Supplement: Figure S4 — DbpA variants produced in B. burgdorferi trigger similar adaptive immune responses. C3H/HeN mice infected with B. burgdorferi strain ML23/pBBE22 (“ML23/Vector”), dbpBA deletion strain ML23ΔdbpBA/pBBE22 (“Vector”), or the deletion strain bearing a plasmid encoding DbpA (“pDbpBA”), DbpAVS461 (“VS461”), DbpAPBr (“PBr”), DbpAN40-D10/E9 (“N40-D10/E9”), or DbpAVS461ΔC11 (“ΔC11”) at doses of 104 spirochetes, were sacrificed at 28 days post-infection. Serum titers of IgG (top panel) and IgM (bottom panel) in mice infected with the indicated strains. Statistical significance was determined using a one-way ANOVA test. Data shown are the mean bacterial loads ± SEM of 8 mice per group. Statistical significance was determined using a one-way ANOVA test. Significant (P<0.05) differences in antibody titers relative to the dbpBA deletion strain (“*”) or relative to the dbpAVS461ΔC11-complemented strain (“†”), are indicated. (TIF) [file ppat.1004238.s004.tif]

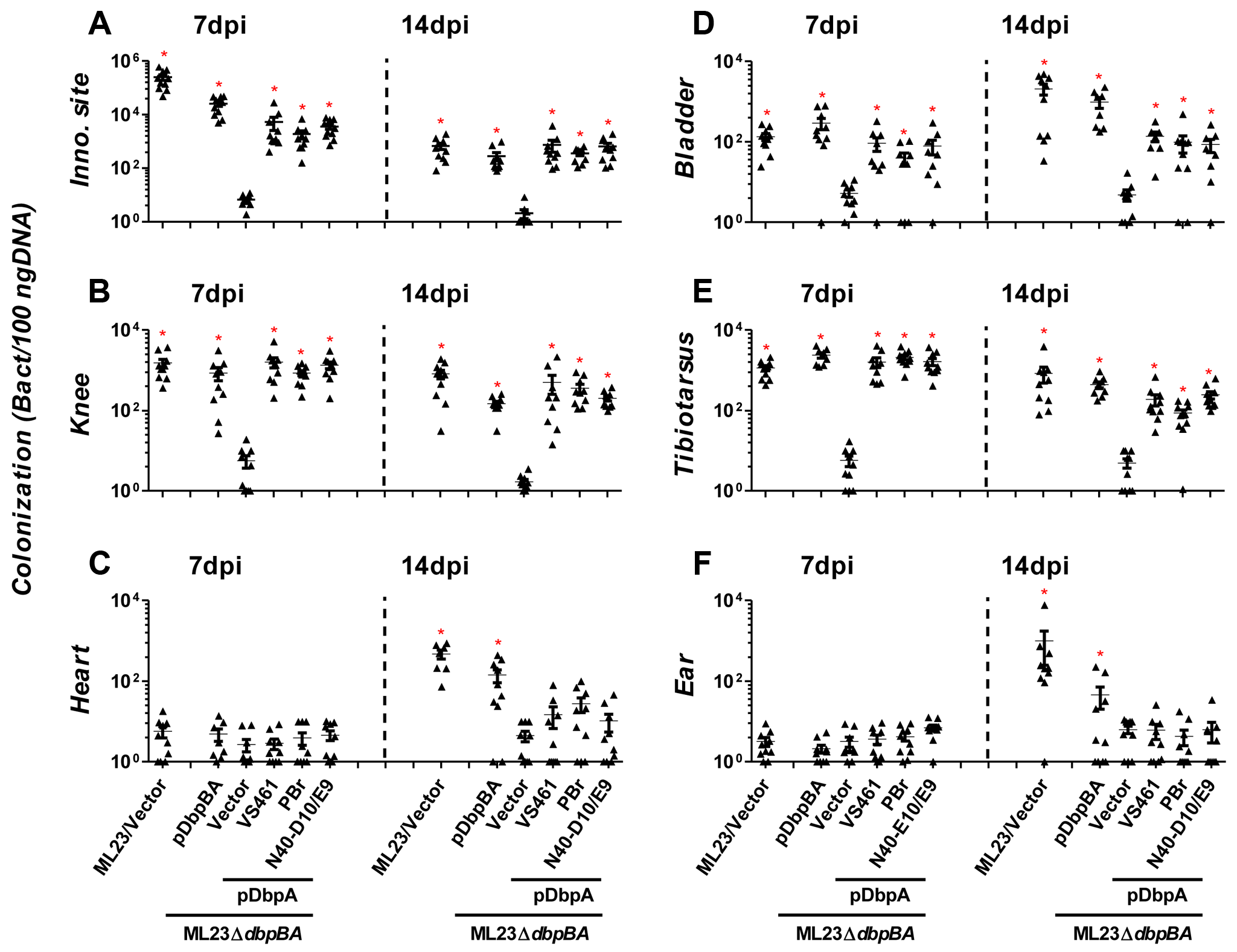

Supplement: Figure S5 — The kinetics of B. burgdorferi dissemination in C3H/HeN mice. C3H/HeN mice infected (104 spirochetes) with B. burgdorferi strain ML23/pBBE22 (“ML23/Vector”), dbpBA deletion strain ML23ΔdbpBA/pBBE22 (“Vector”), or the deletion strain bearing a plasmid encoding the indicated DbpA variants were sacrificed at 7 and 14 days post-infection. Bacterial loads at the (A) inoculation site, (B) knee joint, (C) heart, (D) bladder, (E) tibiotarsus joint, and (F) earwere determined by qPCR. Data shown are the mean bacterial loads ± SEM of 10 mice per group. Statistical significance was determined using a one-way ANOVA test. Significant (P<0.05) differences in spirochete number relative to the dbpBA deletion strain (“*”), or between two strains relative to each other (“#”) are indicated. These data are described comprehensively with other post-infection time points in Table S1. (TIF) [file ppat.1004238.s005.tif]

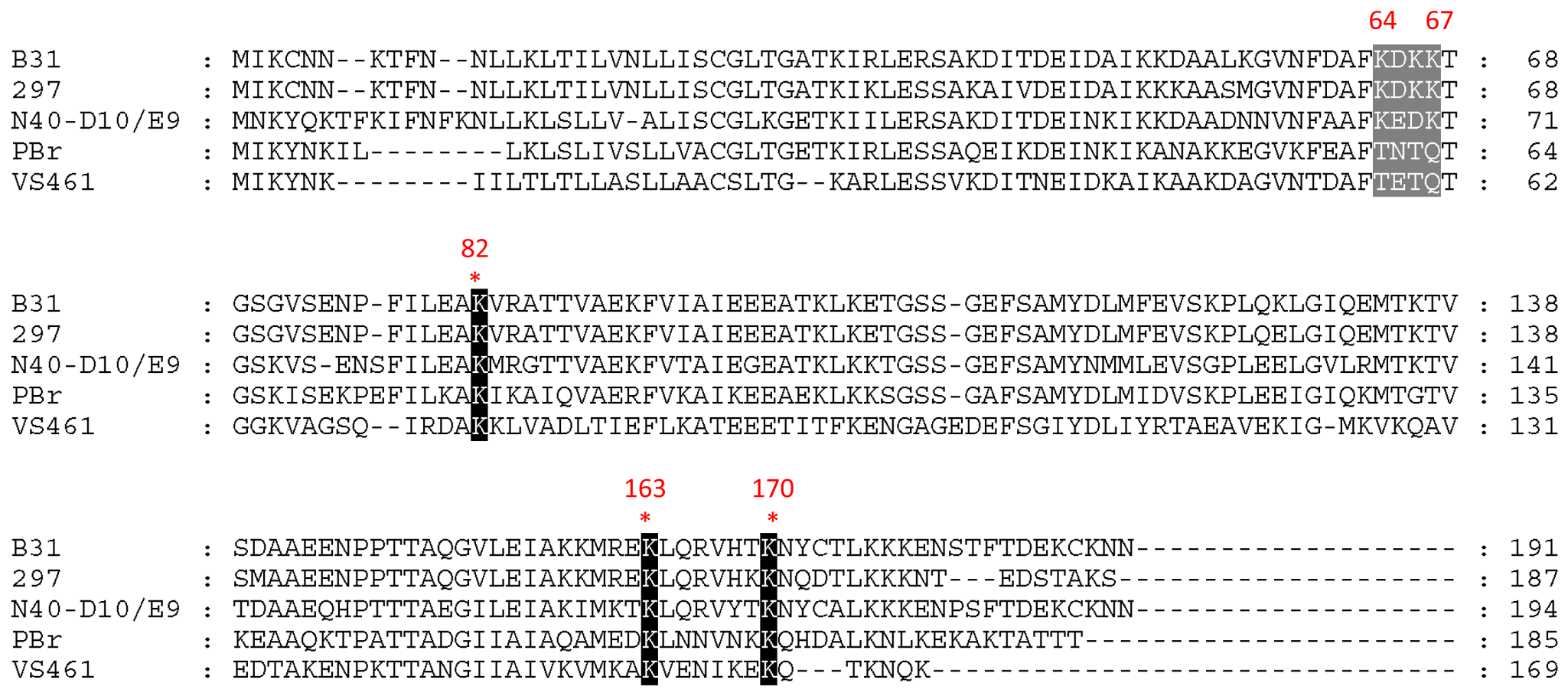

Supplement: Figure S6 — Sequence alignment of DbpA variants found in B31, 297, N40-D10/E9, PBr and VS461 strains for Borrelia . DbpAB356 is not shown because it has 99% sequence identity with strain DbpAN40-D10/E9 [39]. Black shaded residues are the critical residues for decorin- and dermatan sulfate-binding [41]. Gray shaded residues are the residues in BXBB motif previously suggested to be important for decorin- and dermatan sulfate-binding [40], [45]. (TIF) [file ppat.1004238.s006.tif]
