# Supplementary material for: Strain-Specific Variation of the Decorin-Binding Adhesin DbpA Influences the Tissue Tropism of the Lyme Disease Spirochete
Source: PLoS Pathog. 2014 Jul 31;10(7):e1004238. doi: 10.1371/journal.ppat.1004238 (PMC4117581; doi:10.1371/journal.ppat.1004238)
Supplement: Table S1 — Summary of tissue colonization promoted by diverse dbpA alleles. (DOCX) [file ppat.1004238.s007.docx]

**Table S1. Summary of tissue colonization promoted by diverse *dbpA* alleles**

|  |  | Day 3 | Day 7 | Day 14 | Day 21 | Day 28 |
| --- | --- | --- | --- | --- | --- | --- |
| Inoc. site | pDbpBA | 229±70^†^ | 25763±5525^†^ | 287±96 ^†^ | 317±61^†^ | 350±30^†^ |
|  | Vector | 5.4±1.1* | 6.6±0.9* | 2.1±0.7* | 2.3±0.6* | 6.2±1.3* |
|  | pDbpA_VS461_ΔC11 | 3.6±1.0* | ND^a^ | ND^a^ | ND^a^ | 7.9±1.9* |
|  | pDbpA_VS461_ | 31±8*^†^ | 5309±272*^†^ | 750±35 ^†^ | 978±164^†^ | 317±79^†^ |
|  | pDbpA_PBr_ | 171±53^†^ | 1868±631*^†^ | 356±63^†^ | 67±14*^†^ | 33±7* |
|  | pDbpA_N40-D10/E9_ | 18±4*^†^ | 3615±797*^†^ | 665±18^†^ | 466±97^†^ | 145±20^†^ |
|  |  |  |  |  |  |  |
| Bladder | pDbpBA | ND^a^ | 291±90^†^ | 976±25^†^ | 455±16^†^ | 127±28^†^ |
|  | Vector | ND^a^ | 5.3±1.1* | 4.8±1.5* | 5.7±1.1* | 11±2* |
|  | pDbpA_VS461_ΔC11 | ND^a^ | ND^a^ | ND^a^ | ND^a^ | 8.3±2.6* |
|  | pDbpA_VS461_ | ND^a^ | 92±3.3^†^ | 137±31*^†^ | 444±17^†^ | 94±22^†^ |
|  | pDbpA_PBr_ | ND^a^ | 39±6*^†^ | 97±5*^†^ | 242±50^†^ | 49±6*^†^ |
|  | pDbpA_N40-D10/E9_ | ND^a^ | 79±3*^†^ | 85±3*^†^ | 307±73^†^ | 79±10^†^ |
|  |  |  |  |  |  |  |
| Knee | pDbpBA | ND^a^ | 852±30^†^ | 147±20^†^ | 116±40^†^ | 107±33^†^ |
|  | Vector | ND^a^ | 5.5±1.9* | 1.7±0.2* | 4.3±1* | 7.9±2.1* |
|  | pDbpA_VS461_ΔC11 | ND^a^ | ND^a^ | ND^a^ | ND^a^ | ND^a^ |
|  | pDbpA_VS461_ | ND^a^ | 1575±447^†^ | 498±23^†^ | 88±12^†^ | 16±3* |
|  | pDbpA_PBr_ | ND^a^ | 854±13^†^ | 362±87^†^ | 49±10^†^ | 18±4* |
|  | pDbpA_N40-D10/E9_ | ND^a^ | 1328±249^†^ | 200±28^†^ | 212±41^†^ | 86±15^†^ |

|  |  |  |  |  |  |  |
| --- | --- | --- | --- | --- | --- | --- |
| Tibio tarsus | pDbpBA | ND^a^ | 2373±341^†^ | 442±71^†^ | 113±47^†^ | 521±12^†^ |
|  | Vector | ND^a^ | 5.7±1.6* | 4.9±1.2* | 2.9±0.7* | 10±2* |
|  | pDbpA_VS461_ΔC11 | ND^a^ | ND^a^ | ND^a^ | ND^a^ | ND^a^ |
|  | pDbpA_VS461_ | ND^a^ | 1597±405^†^ | 189±59^†^ | 46±15^†^ | 38±11* |
|  | pDbpA_PBr_ | ND^a^ | 2105±285^†^ | 86±18^†^ | 53±23^†^ | 27±18* |
|  | pDbpA_N40-D10/E9_ | ND^a^ | 1620±332^†^ | 244±52^†^ | 81±19^†^ | 202±54^†^ |
|  |  |  |  |  |  |  |
| Heart | pDbpBA | ND^a^ | 2.6±0.9 | 142±50^†^ | 393±96^†^ | 149±41^†^ |
|  | Vector | ND^a^ | 4.8±1.5 | 4.4±1.2* | 6.8±1.7* | 3.4±1.1* |
|  | pDbpA_VS461_ΔC11 | ND^a^ | ND^a^ | ND^a^ | ND^a^ | ND^a^ |
|  | pDbpA_VS461_ | ND^a^ | 4.5±1.2 | 14±8* | 291±11.6^†^ | 19±9* |
|  | pDbpA_PBr_ | ND^a^ | 3.9±1.3 | 27±10* | 1699±552^†^ | 348±96^†^ |
|  | pDbpA_N40-D10/E9_ | ND^a^ | 2.8±0.8 | 10±4* | 36±8.0*^†^ | 6.7±3.1* |
|  |  |  |  |  |  |  |
| Ear | pDbpBA | ND^a^ | 2.1±0.5 | 46±2.6^†^ | 145±21^†^ | 323±91^†^ |
|  | Vector | ND^a^ | 3.2±0.8 | 6.2±1.3* | 8.7±3.4* | 7.3±1.5* |
|  | pDbpA_VS461_ΔC11 | ND^a^ | ND^a^ | ND^a^ | ND^a^ | 9.7±3.1* |
|  | pDbpA_VS461_ | ND^a^ | 3.6±0.9 | 6.1±2.4* | 12.8±5.3* | 1575±671^†^ |
|  | pDbpA_PBr_ | ND^a^ | 4.2±0.9 | 4.3±1.8* | 7.7±2.4* | 101±26^†^ |
|  | pDbpA_N40-D10/E9_ | ND^a^ | 6.9±1.0 | 6.2±3.2* | 12±4* | 293±61^†^ |

Each data point represents the average number ± standard deviation of spirochetes present as determined by qPCR based on dates in Figs. 2, 3, 4, 5, and S5

^a^ Not determined.

*Statistical significance compared with ML23/Vector

^†^ Statistical significance compared with Δ*dbpBA*/Vector
