# Supplementary material for: Strain-Specific Variation of the Decorin-Binding Adhesin DbpA Influences the Tissue Tropism of the Lyme Disease Spirochete
Source: PLoS Pathog. 2014 Jul 31;10(7):e1004238. doi: 10.1371/journal.ppat.1004238 (PMC4117581; doi:10.1371/journal.ppat.1004238)
Supplement: Table S2 — Primers used in this study. (DOCX) [file ppat.1004238.s008.docx]

**Table S2. Primers used in this study.**

| Primer/Vector | Sequence* | Amplified DNA fragment |
| --- | --- | --- |
| DbpA_297_pfp/pQE30 | cgGGATCCgcaacaaaaatcaaatta | *dbpA_297_p* |
| DbpA_297_prp/pQE30 | cgGTCGACttacgatttagcagtgct |  |
| DbpA_B356_pfp/pQE30 | cgGGATCCgaaacaaaaatcatatta | *dbpA_B356_p* |
| DbpA_B356_prp/pQE30 | cgGTCGACttagttatttttgcattt |  |
| DbpA_VS461_∆C11pfp/pQE30 | cgGGATCCagtttaacaggaaaagct | *dbpA_VS461_p* |
| DbpA_VS461_∆C11prp/pQE30 | cgGTCGACttccacttttgctttcat |  |
| pdbpBAfp/pBBE22 | cgGCATGCggcaaactggaaacaagt | *pdbpAB* |
| pdbpBArp/pBBE22 | cgGTCGACtttttcctccttctatta |  |
| DbpA_N40-D10/E9_fp/pBBE22 | cgGTCGACatgaataaatatcaaaaa | *dbpA_N40-D10/E9_* |
| DbpA_N40-D10/E9_rp/pBBE22 | cgGGATCCttagttatttttgcattt |  |
| DbpA_VS461_fp/pBBE22 | cgGTCGACatgattaaatataataaa | *dbpA_VS461_* |
| DbpA_VS461_rp/pBBE22 | cgGGATCCttatttttgatttttagt |  |
| DbpA_PBr_fp/pBBE22 | cgGTCGACatgattaaatataataaa | *dbpA_PBr_* |
| DbpA_PBr_rp/pBBE22 | cgGGATCCttatgtagtagtagcagt |  |
| DbpA_VS461_∆C11fp/pBBE22 | cgGTCGACatgattaaatataataaa | *dbpA_VS461_∆C11* |
| DbpA_VS461_∆C11rp/pBBE22 | cgGGATCCttattccacttttgctttcat |  |
| BBRecAfp | gtggatctattgtattagatgaggctctcg | *BBRecA* |
| BBRecArp | gccaaagttctgcaacattaacacctaaag |  |
| mNidfp | ccagccacagaatcccatcc | *mNidogen* |
| mNidrp | ggacatactctgctgccatc |  |
| Kanfp | atgagccatattcaacgggaa | Kanamycin |
| Kanrp | ttagaaaaactcatcgagcat |  |
| Genfp | atgttacgcagcagcaac | Gentamycin |
| Genrp | ttaggtggcggtacttgg |  |

* The restriction sites used are shown as capital letters.
