# Supplementary material for: Strain-Specific Variation of the Decorin-Binding Adhesin DbpA Influences the Tissue Tropism of the Lyme Disease Spirochete
Source: PLoS Pathog. 2014 Jul 31;10(7):e1004238. doi: 10.1371/journal.ppat.1004238 (PMC4117581; doi:10.1371/journal.ppat.1004238)
Supplement: Table S3 — Bacterial strains and plasmids used in this study. (DOCX) [file ppat.1004238.s009.docx]

**Table S3. Bacterial strains and plasmids used in this study.**

| Strain or Plasmid | Genotype or characteristics | Reference(s) or source |
| --- | --- | --- |
| *B. burgdorferi* strains |  |  |
| B314 | High-passage *B. burgdorferi* B31 missing lp5, lp16, lp17, lp21, lp25, lp28-1, lp28-2, lp28-3, lp28-4, lp36, lp29, lp38, lp49, lp54, lp56, cp9, cp32-6, cp32-7, cp32-9. | [[5](#_ENREF_5)] |
| B314/pJF21 | B314 harboring pJF21 vector | [[6](#_ENREF_6)] |
| ML23 | *B. burgdorferi* strain B31 missing lp25 | [[7](#_ENREF_7)] |
| ML23/pBBE22 | Clonal isolate of *B. burgdorferi* strain B31 lacking lp25 carrying plasmid pBBE22. This plasmid contained bbe22, the nicotinamidase, under the control of a strong borrelial promoter (PflaB). | [[8](#_ENREF_8)] |
| JF105 | ML23 ∆*dbpBA*::GentR^a^ | [[9](#_ENREF_9)] |
| JF/105/pBBE22 | ML23 ∆*dbpBA*::GentR^a^ carrying plasmid pBBE22, which contained bbe22 under the control of a strong borrelial promoter (PflaB) | [[9](#_ENREF_9)] |
| JF105/pJBF17 | ML23 ∆*dbpBA*::GentR^a^ complemented with intact *dbpBA* from *B. burgdorferi* strain B31 under the control of *dbpBA* promoter (PdbpBA) from *B. burgdorferi* strain B31 and bbe22 under the control of a strong borrelial promoter (PflaB). | [[9](#_ENREF_9)] |
| JF105/pDbpA_N40-D10/E9_ | ML23 ∆*dbpBA*::GentR^a^ complemented with *dbpA* from *B. burgdorferi* strain N40-D10/E9 under the control of *dbpBA* promoter (PdbpBA) from *B. burgdorferi* strain B31 and bbe22 under the control of a strong borrelial promoter (PflaB). | This study |
| JF105/pDbpA_VS461_ | ML23 ∆*dbpBA*::GentR^a^ complemented with *dbpA* from *B. afzelii* strain VS461 under the control of *dbpBA* promoter (PdbpBA) from *B. burgdorferi* strain B31 and bbe22 under the control of a strong borrelial promoter (PflaB). | This study |
| JF105/pDbpA_PBr_ | ML23 ∆*dbpBA*::Gent^ra^ complemented with *dbpA* from *B. garinii* strain PBr under the control of *dbpBA* promoter (PdbpBA) from *B. burgdorferi* strain B31 and bbe22 under the control of a strong borrelial promoter (PflaB). | This study |
| JF105/pDbpA_VS461_∆C11 | ML23 ∆*dbpBA*::Gent^ra^ complemented with *dbpA* from *B. afzelii* strain VS461 lacking C-terminal 11 amino acids under the control of *dbpBA* promoter (PdbpBA) from *B. burgdorferi* strain B31 and bbe22 under the control of a strong borrelial promoter (PflaB). | This study |
|  |  |  |
| *E. coli* strains |  |  |
| DH10B | F^-^ *mcr*A Δ(*mrr-hsd*RMS-*mcr*BC) φ80*lac*ZΔM15 Δ*lac*X74 *rec*A1 *end*A1 *ara*D139Δ(*ara, leu*)7697 *gal*U *gal*K λ^-^ *rps*L *nup*G | Invitrogen |
| BL21 | F^–^, *ompT, hsd*SB (rB^–^, mB^–^), *dcm, gal*, λ(DE3) | Promega |
| M15 [pREP4] | *nal^S^ str^S^ rif^S,S^ thi^-^* *lac^-^* *ara^-^* *gal^-^* *mtl^-^* *F^-^* *recA^+^* *uvr^+^* *lon^+^* [ *pREP4 Kan^r^* ] | Qiagen |
| BL21/pET15b-DbpA_B31_ | BL21 expressing DbpA from *B. burgdorferi* strain B31 | This study |
| BL21/pET15b-DbpA_N40-D10/E9_ | BL21 expressing DbpA from *B. burgdorferi* strain N40-D10/E9 | This study |
| BL21/pET15b-DbpA_VS461_ | BL21 expressing DbpA from *B. afzelii* strain VS461 | This study |
| BL21/pET15b-DbpA_PBr_ | BL21 expressing DbpA from *B. garinii* strain PBr | This study |
| M15/pQE30-DbpA_B356_ | M15 expressing DbpA from *B. burgdorferi* strain B356 | This study |
| M15/pQE30-DbpA_297_ | M15 expressing DbpA from *B. burgdorferi* strain 297 | This study |
| M15/pQE30-DbpA_VS461_∆C11 | M15 expressing DbpA from *B. afzelii* strain VS461 lacking C-terminal 11 amino acids | This study |
|  |  |  |
| Plasmids |  |  |
| pCR2.1-TOPO | Amp^rb^, Kan^rc^; PCR cloning vector | Invitrogen |
| pBSV2 | Kan^rc^; borrelial shuttle vector | [[10](#_ENREF_10)] |
| pJF21 | Kan^rc^; pBSV2-derived shuttle vector containing *OspC* promoter | [[6](#_ENREF_6)] |
| pBBE22 | pBSV2 carrying the nicotinamidase (*bbe22/pncA*) under the control of a strong borrelial promoter (PflaB) to restore an infectious phenotype of the strains lacking lp25 | [[8](#_ENREF_8)] |
| pJBF17 | Kan^r^; intact *dbpBA* recombined via the plasmid pBBE22gate, which was modified from pBBE22 by Invitrogen's Gateway recombination-based cloning system. | [[9](#_ENREF_9)] |
| pDbpA_N40-D10/E9_ | pBSV2 carrying *dbpA* from *B. burgdorferi* strain N40-D10/E9 under the control of *dbpBA* promoter (pdbpBA) from *B. burgdorferi* strain B31 and *bbe22* under the control of a strong borrelial promoter (PflaB) | This study |
| pDbpA_VS461_ | pBSV2 carrying *dbpA* from *B. afzelii* strain VS461 under the control of *dbpBA* promoter (pdbpBA) from *B. burgdorferi* strain B31 and *bbe22* under the control of a strong borrelial promoter (PflaB) | This study |
| pDbpA_PBr_ | pBSV2 carrying *dbpA* from *B. garinii* strain PBr under the control of *dbpBA* promoter (pdbpBA) from *B. burgdorferi* strain B31 and *bbe22* under the control of a strong borrelial promoter (PflaB) | This study |
| pDbpA_VS461_∆C11 | pBSV2 carrying *dbpA* from *B. afzelii* strain VS461 lacking C-terminal 11 amino acids under the control of *dbpBA* promoter (pdbpBA) from *B. burgdorferi* strain B31 and *bbe22* under the control of a strong borrelial promoter (PflaB) | This study |

^a^Gent^r^, Gentamycin resistance

^b^Amp^r^, Ampicillin resistance

^c^Kan^r^, Kanamycin resistance
