# Supplementary material for: Strain-Specific Variation of the Decorin-Binding Adhesin DbpA Influences the Tissue Tropism of the Lyme Disease Spirochete
Source: PLoS Pathog. 2014 Jul 31;10(7):e1004238. doi: 10.1371/journal.ppat.1004238 (PMC4117581; doi:10.1371/journal.ppat.1004238)
Supplement: Text S1 — Supplemental references. (DOCX) [file ppat.1004238.s010.docx]

**SUPPLEMENTAL REFERENCES**

1. Benoit VM, Fischer JR, Lin YP, Parveen N, Leong JM (2011) Allelic variation of the Lyme disease spirochete adhesin DbpA influences spirochetal binding to decorin, dermatan sulfate, and mammalian cells. Infect Immun 79: 3501-3509.

2. Brown EL, Guo BP, O'Neal P, Hook M (1999) Adherence of Borrelia burgdorferi. Identification of critical lysine residues in DbpA required for decorin binding. J Biol Chem 274: 26272-26278.

3. Wang X (2012) Solution structure of decorin-binding protein A from Borrelia burgdorferi. Biochemistry 51: 8353-8362.

4. Morgan A, Wang X (2013) The Novel Heparin-Binding Motif in Decorin-Binding Protein A from Strain B31 of Borrelia burgdorferi Explains the Higher Binding Affinity. Biochemistry 52: 8237-8245.

5. Sadziene A, Wilske B, Ferdows MS, Barbour AG (1993) The cryptic ospC gene of Borrelia burgdorferi B31 is located on a circular plasmid. Infect Immun 61: 2192-2195.

6. Fischer JR, LeBlanc KT, Leong JM (2006) Fibronectin binding protein BBK32 of the Lyme disease spirochete promotes bacterial attachment to glycosaminoglycans. Infect Immun 74: 435-441.

7. Labandeira-Rey M, Skare JT (2001) Decreased infectivity in Borrelia burgdorferi strain B31 is associated with loss of linear plasmid 25 or 28-1. Infect Immun 69: 446-455.

8. Purser JE, Lawrenz MB, Caimano MJ, Howell JK, Radolf JD, et al. (2003) A plasmid-encoded nicotinamidase (PncA) is essential for infectivity of Borrelia burgdorferi in a mammalian host. Mol Microbiol 48: 753-764.

9. Weening EH, Parveen N, Trzeciakowski JP, Leong JM, Hook M, et al. (2008) Borrelia burgdorferi lacking DbpBA exhibits an early survival defect during experimental infection. Infect Immun 76: 5694-5705.

10. Stewart PE, Thalken R, Bono JL, Rosa P (2001) Isolation of a circular plasmid region sufficient for autonomous replication and transformation of infectious Borrelia burgdorferi. Mol Microbiol 39: 714-721.
